# Efficacy of the Flo App in Improving Health Literacy, Menstrual and General Health, and Well-Being in Women: Pilot Randomized Controlled Trial

**DOI:** 10.2196/54124

**Published:** 2024-05-02

**Authors:** Adam C Cunningham, Carley Prentice, Kimberly Peven, Aidan Wickham, Ryan Bamford, Tara Radovic, Anna Klepchukova, Maria Fomina, Katja Cunningham, Sarah Hill, Liisa Hantsoo, Jennifer Payne, Liudmila Zhaunova, Sonia Ponzo

**Affiliations:** 1 Flo Health UK Limited London United Kingdom; 2 Maternal, Adolescent, Reproductive & Child Health Centre London School of Hygiene & Tropical Medicine London United Kingdom; 3 Department of Psychology and Ergonomics Technische Universitaet Berlin Berlin Germany; 4 Department of Behavioural Science and Health University College London London United Kingdom; 5 Department of Psychology Texas Christian University Fort Worth, TX United States; 6 Department of Psychiatry & Behavioral Sciences Johns Hopkins University Baltimore, MD United States; 7 Department of Psychiatry and Neurobehavioral Sciences University of Virginia Charlottesville, VA United States; 8 Institute of Health Informatics University College London London United Kingdom

**Keywords:** digital health, health literacy, menstrual cycle, period tracking app, women’s health, PMS, PMDD, tracking, app, application, tracking app, tracking application, menstrual, women, efficacy, general health, wellbeing, randomized controlled trial, awareness, symptoms, manage, management, premenstrual, premenstrual syndrome, premenstrual dysphoric disorder, reproductive, reproductive health, health management, communication, pregnancy, quality of life, productivity, education, functionality

## Abstract

**Background:**

Reproductive health literacy and menstrual health awareness play a crucial role in ensuring the health and well-being of women and people who menstruate. Further, awareness of one’s own menstrual cycle patterns and associated symptoms can help individuals identify and manage conditions of the menstrual cycle such as premenstrual syndrome (PMS) and premenstrual dysphoric disorder (PMDD). Digital health products, and specifically menstrual health apps, have the potential to effect positive change due to their scalability and ease of access.

**Objective:**

The primary aim of this study was to measure the efficacy of a menstrual and reproductive health app, Flo, in improving health literacy and health and well-being outcomes in menstruating individuals with and without PMS and PMDD. Further, we explored the possibility that the use of the Flo app could positively influence feelings around reproductive health management and communication about health, menstrual cycle stigma, unplanned pregnancies, quality of life, work productivity, absenteeism, and body image.

**Methods:**

We conducted 2 pilot, 3-month, unblinded, 2-armed, remote randomized controlled trials on the effects of using the Flo app in a sample of US-based (1) individuals who track their cycles (n=321) or (2) individuals who track their cycles and are affected by PMS or PMDD (n=117).

**Results:**

The findings revealed significant improvements at the end of the study period compared to baseline for our primary outcomes of health literacy (cycle tracking: D̄=1.11; *t*_311_=5.73, *P*<.001; PMS or PMDD: D̄=1.20; *t*_115_=3.76, *P*<.001) and menstrual health awareness (D̄=3.97; *t*_311_=7.71, *P*<.001), health and well-being (D̄=3.44; *t*_311_=5.94, *P*<.001), and PMS or PMDD symptoms burden (D̄=–7.08; *t*_115_=–5.44, *P*<.001). Improvements were also observed for our secondary outcomes of feelings of control and management over health (D̄=1.01; *t*_311_=5.08, *P*<.001), communication about health (D̄=0.93; *t*_311_=2.41, *P*=.002), menstrual cycle stigma (D̄=–0.61; *t*_311_=–2.73, *P*=.007), and fear of unplanned pregnancies (D̄=–0.22; *t*_311_=–2.11, *P*=.04) for those who track their cycles, as well as absenteeism from work and education due to PMS or PMDD (D̄=–1.67; *t*_144_=–2.49, *P*=.01).

**Conclusions:**

These pilot randomized controlled trials demonstrate that the use of the Flo app improves menstrual health literacy and awareness, general health and well-being, and PMS or PMDD symptom burden. Considering the widespread use and affordability of the Flo app, these findings show promise for filling important gaps in current health care provisioning such as improving menstrual knowledge and health.

**Trial Registration:**

OSF Registries osf.io/pcgw7; https://osf.io/pcgw7 ; OSF Registries osf.io/ry8vq; https://osf.io/ry8vq

## Introduction

Many women of childbearing age lack foundational knowledge about their menstrual cycle despite it being a vital indicator of women’s health [1]. Nearly half of all women are unaware of the average length of a regular menstrual cycle and around 40% are unfamiliar with the ovulatory cycle [1,2]. Poor knowledge about menstrual health can hinder women’s ability to make informed decisions about their health [3], negatively impact their daily sociocultural activities [4-6], and contribute to misconceptions and taboos that compromise their overall physical and mental well-being [7,8]. For example, a recent systematic review found that poor knowledge regarding menstrual health may lead to negative experiences such as shame and the lack of confidence to engage in social activities, ultimately increasing mental burden [9]. Further, low menstrual literacy has been associated with inadequate self-care behaviors [10] and reduced satisfaction with medical visits, attributed to ineffective communication between patients and health care providers [11]. This is further evidenced by the results of the UK Women’s Health Strategy Survey, where as many as 1 in 4 women reported not feeling comfortable talking to health care professionals about their menstrual cycle [12]. Interventions aimed at improving menstrual cycle knowledge are therefore likely to be associated with improvements in a wide range of domains relevant to women’s physical and mental health.

A good example of where improved menstrual and reproductive health literacy may have beneficial outcomes is in the case of premenstrual syndrome (PMS) and premenstrual dysphoric disorder (PMDD). PMS and PMDD, a more severe form of PMS, are common, with a worldwide prevalence of 48% for PMS [13,14] and 3% to 8% for PMDD [14]. Affected individuals report a broad range of psychological and physical symptoms occurring in the luteal phase of the menstrual cycle, which can cause social or economic dysfunction and affective and functional impairment [15,16]. Severe PMS symptoms are associated with poorer ratings of quality of life and higher levels of stress [14,17,18]. Similarly, the health burden of PMDD has been deemed greater than type 2 diabetes and hypertension in terms of reported pain [14,18]. Both PMS and PMDD have a proven negative impact on workplace productivity and in some cases even lead to absenteeism [19,20]. Despite this heavy burden on individuals and society, these conditions are often poorly treated and overlooked in research. Evidence suggests that the symptoms of PMS and PMDD may be dismissed by health care professionals, and even by the affected women themselves, who might consider them as a normal part of the menstrual cycle [21,22], highlighting how poor health literacy can result in delays in seeking care for reproductive health conditions [1,23,24].

While there is a notable lack of research into topics related to women’s health [25], there is an acute shortage of research that validates the effectiveness of educational interventions for menstrual health literacy. Most studies have relatively small sample sizes or focus on limited populations or age ranges [26,27]. Digital, internet, and mobile-based health technologies can provide novel ways to deliver interventions for menstrual health and have gained popularity due to their scalability, availability, and anonymity [28,29], with the latter being crucial for menstrual and mental health [30-35]. Health apps focusing on menstrual and reproductive health can help individuals feel more prepared for their menstrual cycle symptoms, be more aware of their body signals throughout the cycle, and be better able to manage associated symptoms [36]. Additionally, evidence suggests that self-guided interventions using mobile phones can successfully equip individuals experiencing PMS and PMDD with strategies to promote and maintain healthy behavior, improve PMS symptoms, and reduce their interference with everyday life activities [37,38]. Although preliminary studies show promising results, research on the efficacy of such health solutions remains limited.

The Flo smartphone app [39] (Flo Health) is a period tracking and reproductive health app. It provides personalized menstruation and ovulation predictions, symptom forecasts based on user input, and expert-reviewed content accessible via the library or the health assistant (chatbot). The users of the Flo app also interact anonymously on a community platform called “Secret Chats,” which facilitates conversations on stigmatized topics such as sexual and menstrual health [29,30,33].

The primary objective of these 2 pilot randomized controlled trials (RCTs) was to measure the efficacy of the Flo app in improving menstrual-related health literacy and awareness and well-being outcomes in (1) individuals who reported an interest in using the app to track their cycles and symptoms (trial 1) or (2) individuals who reported an interest in using the app to track their cycles and symptoms and reported being affected by symptoms of conditions of the menstrual cycle such as PMS and PMDD (trial 2). The secondary objective was to investigate the effect of app use in these populations on overall feelings of health management, communication about health (eg, with health care providers or with their partner), menstrual cycle stigma, fear of unplanned pregnancies, quality of life, productivity at work, absenteeism, and body image. For trial 1, we hypothesized that the use of the Flo app would significantly improve menstrual-related health literacy and awareness and general health and well-being outcomes in the interventional group compared to the waitlist control group; for trial 2, we hypothesized that PMS- or PMDD-related health literacy and PMS or PMDD symptom burden would significantly decrease in the interventional group compared to the waitlist control group. We also hypothesized that all secondary outcomes would either significantly improve (feelings of health management, communication about health with health care providers or with a partner, quality of life, productivity at work, and body image) or decrease (menstrual cycle stigma, fear of unplanned pregnancies, and absenteeism from work or school due to PMS or PMDD symptoms) compared to the waitlist control group.

## Methods

### Study Design

We intended to conduct 2 fully powered, 3-month, unblinded, 2-armed RCTs, with a between-subjects component (group: intervention or control) and a within-subjects component (time point; see Multimedia Appendix 1 for the CONSORT [Consolidated Standards of Reporting Trials] checklist).

Trial 1 aimed to investigate the efficacy of the Flo app in improving health literacy and health and well-being outcomes in participants who report their current reproductive health aim as (1) cycle tracking, (2) trying to conceive (TTC), or (3) pregnancy tracking. Trial 2 aimed to investigate the efficacy of the Flo app in improving health literacy and PMS and PMDD symptom burden outcomes in participants who report moderate-to-severe PMS or PMDD symptoms. Trials 1 and 2 have been registered on the Open Science Framework [40,41].

Due to recruitment issues in trial 1, very low numbers of eligible participants were recruited in the pregnancy tracking group, and an extremely low number of participants completed the study in the TTC group. We therefore deviated from the preregistered study design by excluding these groups from the further analyses and focused only on the cycle tracking group. Additionally, as both trials had relatively low numbers of study participants who followed the study protocol, we converted our trials into feasibility pilots to inform further full RCTs with improved recruitment strategies. All questionnaire measures were self-reported through a web-based questionnaire platform (Survey Monkey).

### Ethical Considerations

Trials 1 and 2 were approved by an independent ethical review board (WCG IRB, ethics applications 20222535 and 20222597, respectively), using protocols specific to each population. All participants provided informed, electronic consent via a checkbox prior to their enrollment in the study, and data were deidentified. Participants who fully completed all data collection activities received a free yearly subscription to Flo Premium (US $39.99) and were entered into a lottery to win up to US $600 in Amazon Vouchers. This study was funded by Flo Health UK LTD. The authors assert that all procedures contributing to this work comply with the ethical standards of the relevant national and institutional committees on human experimentation and with the Helsinki Declaration of 1975, as revised in 2008.

### Recruitment

For both trials, computer and internet-literate participants were recruited using participant pools (SurveySwap, PureSpectrum, and Prolific) as well as via social media and mailing lists. Recruitment took place between July and December 2022, and potential participants were screened for eligibility via screening SurveyMonkey surveys (screening stage). It may have been possible for the same participant to respond under different email addresses, but these addresses would also need to be registered to the Flo app.

Inclusion criteria for trial-1 participants included being aged 18-40 years, being a US resident, being fluent in English, not being a current or former user of Flo app, having a cycle tracking health goal, scoring less than 80% on the cycle tracking health literacy quiz (see the *Measures* section), and scoring less than 80% on the general health and well-being survey (see the *Measures* section). Participants were excluded from trial 1 if they were currently using any form of hormonal contraception (eg, pill, injection, implant, and hormonal coil) as these would influence or stop their natural menstrual cycle. Participants in trial 1 were additionally excluded if they became pregnant during the study period.

Participants were included in trial 2 if they were older than 18 years, scored at least moderate on the Premenstrual Screening Tool (PSST) [42] (see the *Measures* section), scored less than 80% on the PMS or PMDD health literacy quiz (see the *Measures* section), and had never used Flo before. Further, participants were excluded from trial 2 if they were currently taking antidepressants or were currently or had previously received psychiatric treatment or had been diagnosed with a psychiatric disorder other than PMDD at the time of recruitment.

Participants were excluded from any trial if they had their last period more than 90 days prior or were pregnant at the time of recruitment.

### Randomization and Blinding

If participants met inclusion criteria at the screening stage, they were then randomized by minimization using a Python script based on the following factors: cycle tracking or PMS or PMDD health literacy quiz scores, age, yearly household income in US $, highest level of education completed, race or ethnicity, and reproductive health disorders (Multimedia Appendix 2). Trial-1 participants were additionally minimized on general health and well-being scores, along with the number of children and number of pregnancies that did not result in a live birth, to control for differences in health literacy and awareness that might result from previous experience of conception and pregnancy. Trial-2 participants were additionally minimized on PSST score and current day of cycle, to attempt to control for differences in PMS or PMDD symptom burden due to the menstrual cycle phase.

**Figure 1:**
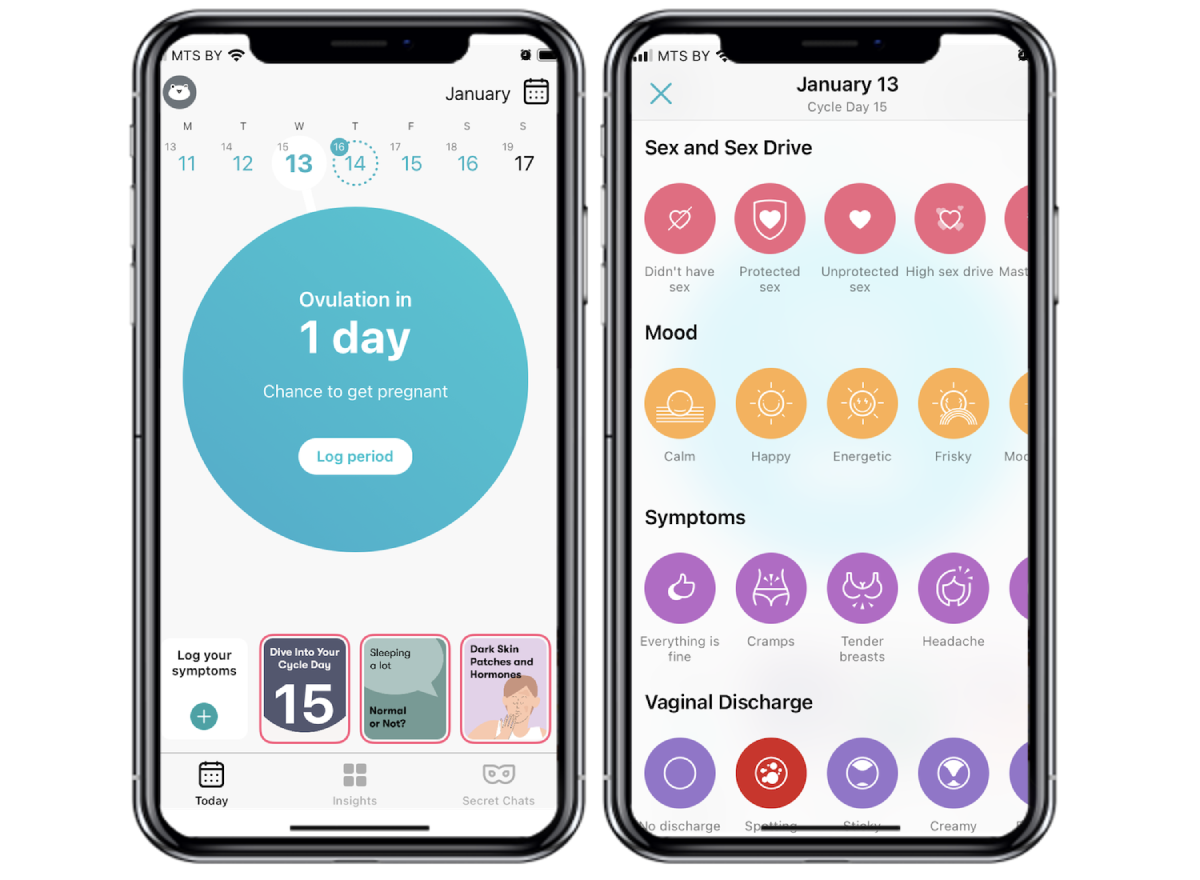
Example of the Flo user interface including the main screen, tiles linking to educational content, cycle day information, and the symptom logging interface.

Participants were either allocated to the trial-specific intervention condition and instructed to use the Flo Premium app for 12 weeks (trial 1) and 3 months (trial 2) or to the waitlist control condition, which received the intervention at the end of the study. An example of the Flo app user interface is shown in Figure 1.

### Intervention Groups

Both trial participants were provided with detailed tutorials via email on how to use the Flo app and instructed to interact freely with the app for the whole duration of the study. All participants were provided with the premium version of the app for 1 year. In comparison to the free offering, premium app users have access to the full range of in-app educational content, all symptom- and cycle-related chatbots, as well as all community features.

### Waitlist Control Groups

Participants who were randomized into the control group were not provided with access to the Flo app during the study period and were instructed not to use the Flo app in any form for the duration of the study. At the end of the study, control participants were provided with the premium version of the app for 1 year.

### Measures

#### Demographics

Demographic information for both trials was collected using a demographic questionnaire, which asked about previous Flo use, US residency, age, race or ethnicity, household income and composition, education, gender identity, reproductive health, contraception use, and timing of last period (Multimedia Appendix 3). Trial-1 participants were additionally asked about English proficiency, parity, prior pregnancy outcomes, and reproductive health goals (cycle tracking, TTC, or pregnancy). Trial-2 participants were additionally asked about employment, present pregnancy status, use of antidepressants, diagnosis of a psychiatric disorder, past or present receival of any treatment for a psychiatric disorder, and perimenopause or menopause symptoms.

#### Primary Outcomes

##### Menstrual and PMS- or PMDD-Related Health Literacy

Menstrual-related health literacy was examined in trial-1 participants using a set of 12 questions regarding general knowledge about the menstrual cycle, such as typical bleeding length, how the length of the menstrual cycle is calculated, and how menstrual symptoms such as pain can be relieved (Multimedia Appendix 4). Knowledge about PMS or PMDD in trial-2 participants was measured through a set of questions investigating PMS and PMDD incidence, symptoms, and methods that can be used to help manage symptoms (Multimedia Appendix 5). The questions used were developed internally by Flo, using input from Flo Health’s medical advisors.

##### Self-Reported Menstrual Health Awareness

Menstrual health awareness was only examined in the trial-1 participants using 7 questions selected from the 33-item Health Literacy Instrument for Adults [43] and modified to be specifically relevant to cycle tracking (Multimedia Appendix 6).

##### Self-Reported General Health and Well-Being

The trial-1 participants answered a set of 14 questions, selected both from domain 2 of the World Health Organization Quality of Life Brief Survey [44] and designed internally by Flo, using input from Flo Health’s medical advisors. These questions assessed self-reported physical, mood, and menstrual symptoms experienced over the previous 3 months, as well as satisfaction with overall health, sex life, personal relationships, and support from friends (Multimedia Appendix 7).

##### PMS and PMDD Symptom Burden

PMS and PMDD symptom burden and the extent to which PMS or PMDD symptoms interfere with daily life were only assessed in trial-2 participants using the PSST [42] (Multimedia Appendix 8).

#### Secondary Outcomes

##### Overview

Feelings of control and management over health, communication and emotional well-being, menstrual cycle stigma (Menstrual Attitude Questionnaire [45]), fear of unplanned pregnancy, and body image and appreciation (Body Appreciation Scale-2) [46] were assessed (Multimedia Appendix 9) in trial-1 participants. Absenteeism due to PMS or PMDD symptoms, work productivity using the Stanford Presenteeism Scale-6 [47], and quality of life and satisfaction using the Quality of Life Enjoyment Satisfaction Questionnaire [48] were additionally assessed in trial-2 participants (Multimedia Appendix 10).

##### App Engagement

Data on app use by the intervention groups were also calculated, including the mean total number of sessions (log-ins), the mean length of sessions in seconds, and the mean number of days of app use (active days).

#### Data Collection Time Points

**Figure 2:**
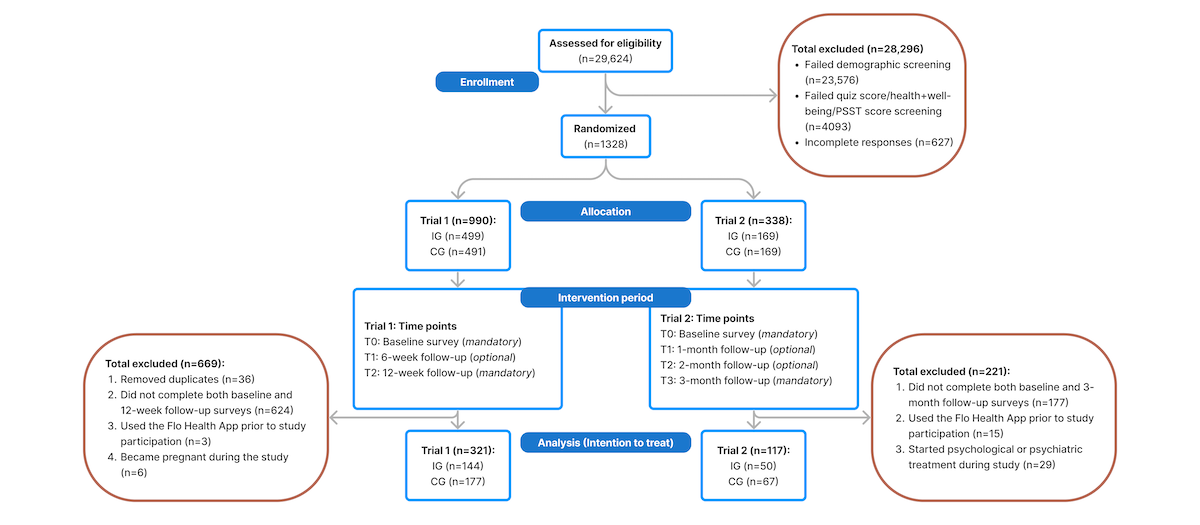
Participant flow diagram through trial 1 (cycle tracking) and trial 2 (premenstrual syndrome or premenstrual dysphoric disorder) intention-to-treat analysis. IG: intervention group; CG: control group; PSST: Premenstrual Screening Tool.

Trial-1 (cycle tracking) participants responded to questionnaires at 3 time points: screening or baseline, 6 weeks after baseline, and 12 weeks after baseline (Figure 2). The screening survey included demographic information, menstrual health literacy, and general health and well-being questions. The baseline and 6-week surveys included menstrual health awareness and all cycle tracking secondary outcome measures. The 12-week survey included the menstrual health literacy, general health and well-being questions, and all cycle tracking secondary outcome measures.

Trial-2 (PMS or PMDD) participants responded to survey questions at screening or baseline, 1 month after baseline, 2 months after baseline, and 3 months after baseline (Figure 2). The screening questionnaire included demographic information and primary outcomes. The baseline survey included all secondary outcome measures, while the month-1 and month-2 questionnaires included PMS or PMDD symptom burden, as well as all secondary outcome measures. The month-3 questionnaire included all primary and secondary outcome measures.

The difference in data collection time points between trials 1 and 2 was due to the need to measure PMS or PMDD symptoms monthly in trial 2.

### Statistical Analyses

#### Data Exclusion

Any participant who did not meet the inclusion criteria based on responses from the screening survey was excluded from the study. To reduce the effect of bias due to noncompliance, we aimed to conduct an intention-to-treat (ITT) analysis and a per-protocol (PP) analysis. In the ITT analysis, participants were analyzed according to the group (intervention or control) they were randomized to, as long as the inclusion criteria were met and the screening, baseline, and final surveys were completed. In the PP analysis, all participants who completed all data collection waves and used the app at least once a month throughout the study duration were included.

#### Data Analysis

Main analysis of all outcomes was conducted using a Mixed Models for Repeated Measures (MMRM) approach. Models were constructed using an unstructured correlation where the outcome of interest was predicted by time point and condition status, including the interaction between time point and condition. Age, highest education level completed, and household income were included as fixed effects. Individual ID and time point were included as random effects. Preplanned pairwise contrasts between baseline outcome scores and final time point scores were used to assess change in outcome scores.

All data analyses were conducted using statistical and data science packages in R (version 4.3.0l R Foundation for Statistical Computing) [49] running on Mac OS X 13.3.1. Packages used included *arsenal* version 3.6.3 [50], *emmeans* version 1.8.6 [51], *ggpubr* version 0.6.0 [52], *gtools* version 3.9.4 [53], *gtsummary* version 1.7.1 [54], *longpower* version 1.0.24 [55-60], *mmrm* version 0.2.2 [61], *patchwork* version 1.1.2 [62], and *tidyverse* version 2.0.0 [63].

#### Power

Using MMRM analysis with 4 data collection waves as is the case in the PMS or PMDD group, an attrition rate of 10% per wave, and a SD of the outcome of interest of 2.5 points, the minimum sample size required to detect a change in score of 1 point with 80% power would be 126 participants in each group. Using 3 data collection waves as in the cycle tracking group, 115 participants assigned to each condition would be required. Due to difficulties in recruiting samples to meet these target group sizes, we converted both trials from full RCTs to feasibility pilot trials.

## Results

### Baseline Sample Characteristics

A total of 438 participants (194 intervention and 244 control) were included in the ITT sample, of which 321 were included in trial 1 (cycle tracking; 144 intervention and 177 control) and 117 were included in trial 2 (PMS or PMDD; 50 intervention and 67 control; Figure 2). The PP sample was comprised of 233 participants (43 intervention and 190 control), of which 174 were included from trial 1 (17 intervention and 157 control) and 59 were included from trial 2 (26 intervention and 33 control; Multimedia Appendix 11). Due to a low number of participants completing all protocol steps, and therefore being eligible for the PP analysis, we present the results and interpretation of the ITT analysis in the main text and avoid interpretation of our underpowered PP results. However, the results of the PP analyses can be found in Multimedia Appendices 8-17.

In the ITT trial-1 and trial-2 samples, we found no differences in demographic characteristics (including age, income, education, or race or ethnicity). At baseline, PMS- or PMDD-related health literacy scores were higher in the intervention group in trial 2. No other primary outcome measures differed between groups in either trial at baseline (Table 1). Demographic and baseline measures for the PP analyses are presented in Multimedia Appendix 12.

Participants in the ITT trial-1 intervention group had a mean session length of 234.45 (SD 181.98) seconds, a mean total number of sessions of 29.94 (SD 20.94), and a mean number of active days of 15.44 (SD 10.95). Participants in the ITT trial-2 intervention group had a mean session length of 146.98 (SD 122.85) seconds, a mean total number of sessions of 70.35 (SD 55.63), and a mean number of active days of 36.08 (SD 24.50). App engagement statistics for the PP sample are shown in Multimedia Appendix 13.

The mean time between completing the screening and baseline surveys was 8.56 (SD 9.86) days in the ITT trial 1 analysis and 9.45 (SD 9.18) days in the ITT trial 2 analysis.

**Table 1:** Intention-to-treat analysis demographic and summary statistics for primary outcome measures at baseline.

Characteristics		Trial 1 (cycle tracking)										Trial 2 (PMS^a^ or PMDD^b^)									
		Control (n=177)		Intervention (n=144)		Total (N=321)		*P* value			Control (n=67)			Intervention (n=50)			Total (N=117)			*P* value	
**Age group (years), n (%)**									.82^c^												.26^c^
	18-24	18 (10.2)	14 (9.7)		32 (10)					9 (13.4)			7 (14)			16 (13.7)					
	25-34	81 (45.8)	71 (49.3)		152 (47.4)					16 (23.9)			20 (40)			36 (30.8)					
	35-44	78 (44.1)	59 (41)		137 (42.7)					33 (49.3)			17 (34)			50 (42.7)					
	45-54	0 (0)	0 (0)		0 (0)					9 (13.4)			6 (12)			15 (12.8)					
**Race or ethnicity, n (%)**									.43^d^												.86^d^
	American Indian or Alaskan Native	2 (1.1)	5 (3.5)		7 (2.2)					3 (4.5)			0 (0)			3 (2.6)					
	Asian or Asian American	8 (4.5)	9 (6.2)		17 (5.3)					5 (7.5)			2 (4)			7 (6)					
	Biracial or multiracial	10 (5.6)	4 (2.8)		14 (4.4)					3 (4.5)			3 (6)			6 (5.1)					
	Black or African American	30 (16.9)	20 (13.9)		50 (15.6)					6 (9)			7 (14)			13 (11.1)					
	Hispanic, Latino, or Spanish origin	14 (7.9)	12 (8.3)		26 (8.1)					9 (13.4)			6 (12)			15 (12.8)					
	Native Hawaiian or Other Pacific Islander	1 (0.6)	0 (0)		1 (0.3)					0 (0)			0 (0)			0 (0)					
	White, European American, or Caucasian	110 (62.1)	94 (65.3)		204 (63.6)					41 (61.2)			32 (64.0)			73 (62.4)					
	Other	2 (1.1)	0 (0)		2 (0.6)					0 (0)			0 (0)			0 (0)					
**Household income (US $), n (%)**									.36^c^												.20^c^
	Missing data	1 (0.6)	5 (3.5)		6 (1.9)					0 (0)			0 (0)			0 (0)					
	Under $15,000	30 (17)	20 (14.4)		50 (15.9)					4 (6.0)			6 (12)			10 (8.5)					
	Between $15,000 and $29,999	33 (18.8)	22 (15.8)		55 (17.5)					9 (13.4)			8 (16)			17 (14.5)					
	Between $30,000 and $49,999	40 (22.7)	46 (33.1)		86 (27.3)					14 (20.9)			10 (20)			24 (20.5)					
	Between $50,000 and $74,999	37 (21)	16 (11.5)		53 (16.8)					18 (26.9)			14 (28)			32 (27.4)					
	Between $75,000 and $99,999	23 (13.1)	13 (9.4)		36 (11.4)					10 (14.9)			6 (12)			16 (13.7)					
	Between $100,000 and $150,000	7 (4)	17 (12.2)		24 (7.6)					9 (13.4)			4 (8)			13 (11.1)					
	Over $150,000	6 (3.4)	5 (3.6)		11 (3.5)					3 (4.5)			2 (4)			5 (4.3)					
**Highest education level, n (%)**									.30^c^												.60^d^
	Missing data	2 (1.1)	0 (0)		2 (0.6)					0 (0)			0 (0)			0 (0)					
	Incomplete or complete secondary education	90 (51.4)	62 (43.1)		152 (47.6)					27 (40.3)			17 (34)			44 (37.6)					
	Some postsecondary education, certificate, or associate degree	29 (16.6)	33 (22.9)		62 (19.4)					10 (14.9)			15 (30)			25 (21.4)					
	Bachelor’s degree or further education	56 (32)	49 (34)		105 (32.9)					30 (44.8)			18 (36)			48 (41)					
**Health literacy**									.90^e^												.03^e^
	Mean (SD)	9.045 (2.205)	9.014 (2.109)		9.031 (2.159)					8.866 (2.014)			9.680 (1.789)			9.214 (1.956)					
	Range	3-14	2-13		2-14					4-12			6-12			4-12					
**Menstrual awareness**									.91^e^												N/A^f^
	Mean (SD)	34.712 (6.178)	34.632 (5.926)		34.676 (6.057)					N/A			N/A			N/A					
	Range	13-49	16-49		13-49					N/A			N/A			N/A					
**Health and well-being**									.25^c^												N/A
	Mean (SD)	44.689 (10.398)	45.972 (9.206)		45.265 (9.887)					N/A			N/A			N/A					
	Range	16-67	19-64		16-67					N/A			N/A			N/A					
**PSST^g^ score**									N/A												.01^e^
	Mean (SD)	N/A	N/A		N/A					33.119 (9.338)			37.280 (7.874)			34.897 (8.948)					
	Range	N/A	N/A		N/A					9-54			15-54			9-54					

^a^PMS: premenstrual syndrome.

^b^PMDD: premenstrual dysphoric disorder.

^c^Trend test for ordinal variables.

^d^Pearson chi-square test.

^e^Linear model ANOVA.

^f^N/A: not applicable.

^g^PSST: Premenstrual Screening Tool.

### Primary Outcomes

#### Menstrual Health Literacy (Trial 1)

**Figure 3:**
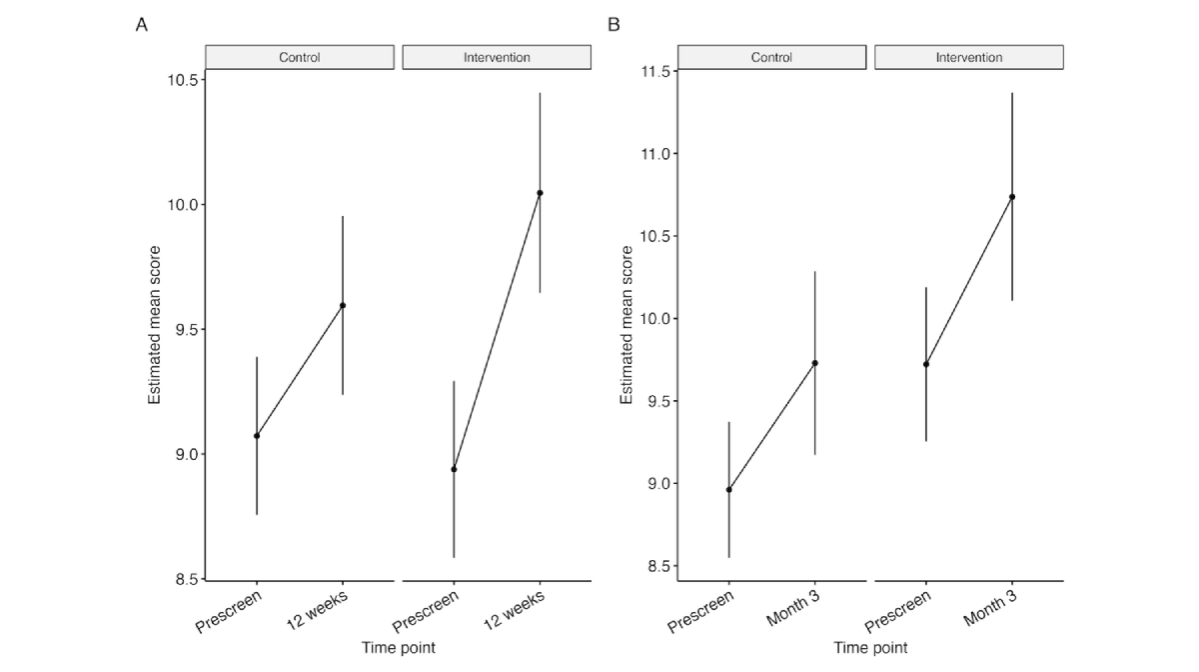
Estimated mean health literacy scores and 95% CIs from Mixed Models for Repeated Measures analysis at screening and end of intervention for the (A) intention-to-treat trial 1 (cycle tracking) and (B) intention-to-treat trial 2 (PMS or PMDD). PMDD: premenstrual dysphoric disorder; PMS: premenstrual syndrome.

Our MMRM analysis in the ITT trial-1 sample revealed significant main effects of time point (β=.523; *P*=.003) and highest education level (β=.488; *P*=.001) on menstrual health literacy, such that menstrual health literacy scores were higher postintervention and in individuals with higher levels of education (Table S1 in Multimedia Appendix 14). We found a significant group by time point interaction (β=.585; *P*=.02), such that greater improvement in menstrual health literacy was observed in the intervention group compared to the control group, with an estimated mean improvement between prescreen and the end of the intervention of 1.1 (SE 0.193) points in the intervention group and to 0.52 (SE 0.173) in the control group (Figure 3A; Table 2). There were no significant associations between menstrual health literacy scores and age group or household income. Similar improvements in estimated mean health literacy scores were seen in the PP analysis (Multimedia Appendix 15).

**Table 2:** Estimated mean differences in Menstrual Health literacy in the ITT^a^ trial-1 analysis (cycle tracking) and PMS^b^ or PMDD^c^ health literacy scores in the ITT trial-2 analysis (PMS or PMDD) between baseline and the end of the intervention.

Trial	Control or intervention	Estimated mean difference (SE)	*T* ratio (*df*)	*P* value
Trial 1 (cycle tracking)	Control	0.523 (0.173)	3.026 (311)	.003
Trial 1 (cycle tracking)	Intervention	1.108 (0.193)	5.729 (311)	<.001
Trial 2 (PMS or PMDD)	Control	0.642 (0.276)	2.327 (115)	.02
Trial 2 (PMS or PMDD)	Intervention	1.200 (0.319)	3.758 (115)	<.001

^a^ITT: intention-to-treat.

^b^PMS: premenstrual syndrome.

^c^PMDD: premenstrual dysphoric disorder.

#### PMS- or PMDD-Related Health Literacy (Trial 2)

Estimated mean PMS or PMDD health literacy score also improved in both the ITT trial-2 intervention and control groups between baseline and end of intervention (Figure 3B; Table 2). We observed significant main effects of the highest education level (β=.430; *P*=.04), condition (β=.819; *P*=.03), and time point (β=.642; *P*=.02; Table S2 in Multimedia Appendix 14). Individuals with higher education had higher average health literacy scores, participants in the intervention group had higher average health literacy scores than controls at both time points, and health literacy scores increased after the intervention in both groups. No association between household income or age group and PMS or PMDD health literacy score was found. Estimated mean health literacy scores increased by 1.2 (SE 0.319) points in the intervention group compared to 0.64 (SE 0.276) in the control group (Table 2). Similar improvements were found in the PP analysis (Multimedia Appendix 15).

#### Self-Reported Menstrual Health Awareness (Trial 1)

**Figure 4:**
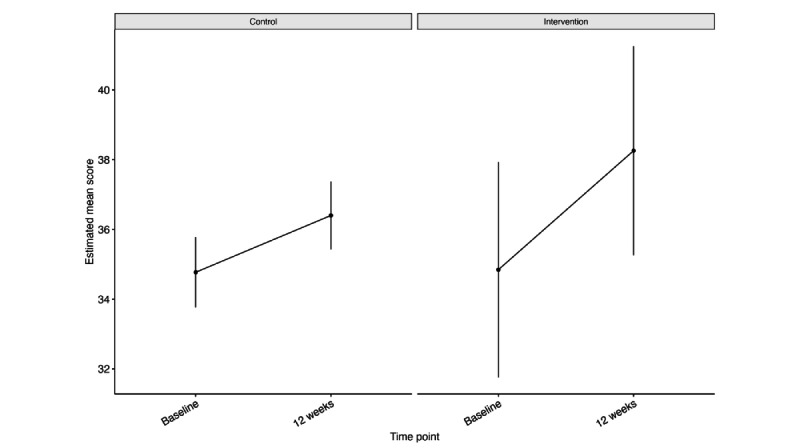
Estimated mean self-reported menstrual health awareness scores and 95% CIs from ITT MMRM analysis at baseline and 12 weeks after baseline for trial 1 (cycle tracking).

In the ITT trial-1 analysis, we observed significant associations between menstrual health awareness and age group (β=1.053; *P*=.03) and time point (β=1.845; *P*<.001; Table S3 in Multimedia Appendix 14), where individuals in the older age groups had higher menstrual health awareness scores, and menstrual health awareness scores were higher after the intervention. We also found a significant interaction between treatment group and time point (β=2.126; *P*=.002), indicating that individuals in the intervention group had a greater improvement in menstrual health awareness scores (Figure 4). No association was found between highest education level, household income or treatment group, and menstrual health awareness scores. Estimated mean scores increased by around 4 (SE 0.515) points in the intervention group, as compared to 1.8 (SE 0.461) points in the control group (Table 3). Similar improvements in menstrual health awareness were observed in the PP analysis (Multimedia Appendix 15).

**Table 3:** Estimated mean differences in menstrual health awareness from trial 1 (cycle tracking).

Control or intervention	Estimated mean difference (SE)	*T* ratio (*df*=311)	*P* value
Control	1.845 (0.461)	4.005	<.001
Intervention	3.971 (0.515)	7.706	<.001

#### Self-Reported General Health and Well-Being (Trial 1)

**Figure 5:**
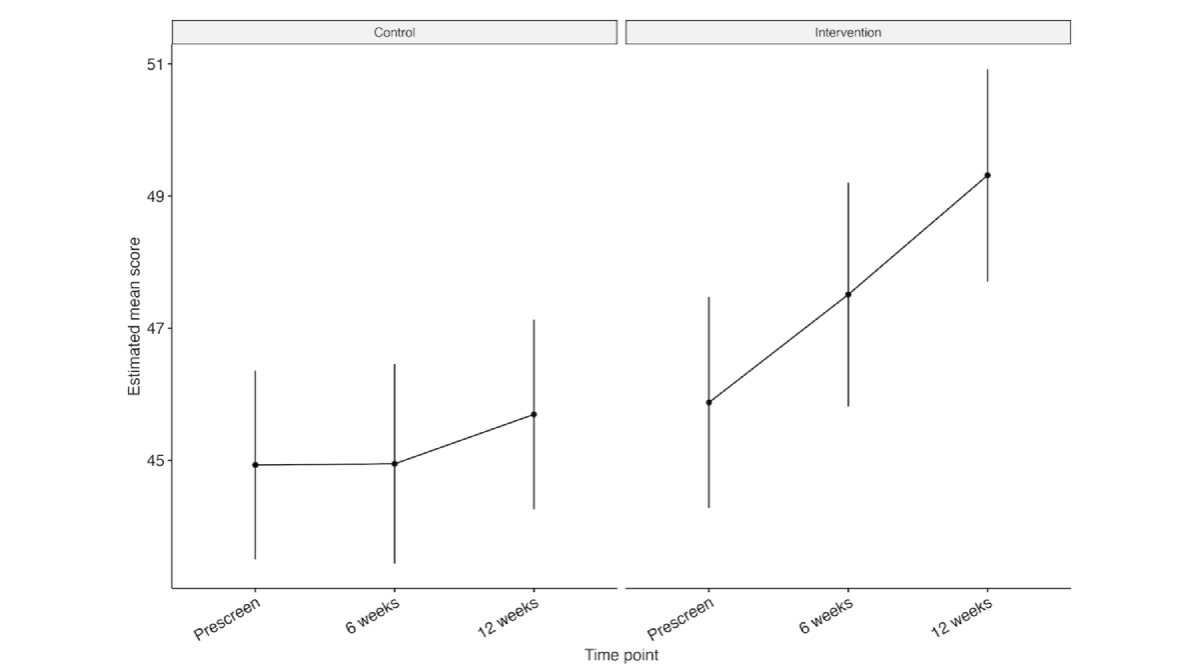
Estimated mean general health and well-being scores and 95% CIs from intention-to-treat Mixed Models for Repeated Measures approach analysis at screening, 6 weeks, and 12 weeks after baseline for trial 1 (cycle tracking).

Our ITT MMRM analysis revealed significant associations between household income and general health and well-being scores (β=1.444; *P*<.001; Table S4 in Multimedia Appendix 14), with individuals with higher income having better health and well-being scores. We found significant interactions between the treatment group and the 6-week time point (β=1.618; *P*=.03) and treatment group and 12-week time point (β=2.674; *P*=.001), indicating at both time points that individuals in the intervention group had greater improvement in health and well-being scores than in the control group. No association was found between age group, treatment group, or 6- and 12-week time points, and health and well-being scores. Estimated mean health and well-being scores improved by 3.44 (SE 0.579) points in the intervention group as compared to 0.76 (SE 0.517) points in the control group, with a significant improvement in estimated mean health and well-being scores between screening and the end of intervention observed in the intervention group alone (Figure 5; Table 4). Improvements in health and well-being scores were found in the intervention condition only in the PP analysis (Multimedia Appendix 15).

**Table 4:** Estimated mean differences in general health and well-being scores between screening and 12 weeks after baseline for the ITT^a^ trial-1 (cycle tracking) analysis.

Control or intervention	Estimated mean difference (SE)	*T* ratio (*df*=311)	*P* value
Control	0.764 (0.517)	1.478	.14
Intervention	3.439 (0.579)	5.944	<.001

^a^ITT: intention-to-treat.

#### PMS or PMDD Symptom Burden (Trial 2)

**Figure 6:**
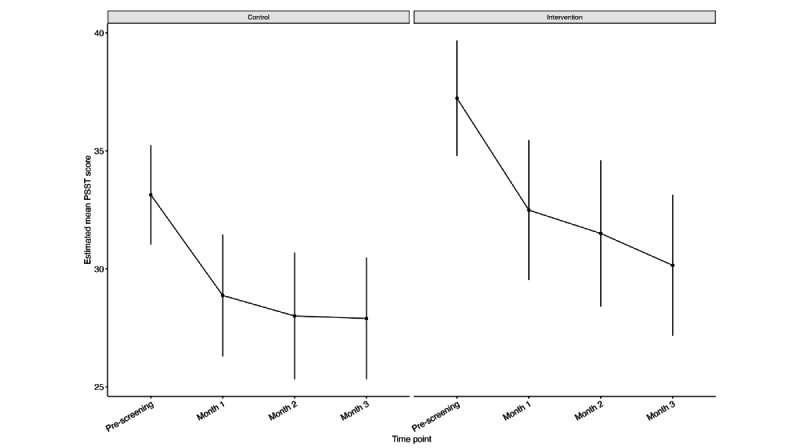
Intention-to-treat Mixed Models for Repeated Measures estimated mean Premenstrual Screening Tool scores at each timepoint for intention-to-treat trial 2 (premenstrual syndrome or premenstrual syndrome).

We found significant main effects of group (β=4.094; *P*=.014), month-1 time point (β=–4.246; *P*<.001), month-2 time point (β=–5.133; *P*<.001), and month-3 time point (β=–5.239; *P*<.001; Table S5 in Multimedia Appendix 14). There were no significant interactions, and no associations were found between age group, highest education, household income, or treatment group with PMS or PMDD symptom burden. Estimated mean PSST scores improved by 7.1 (SE 1.300) points in the intervention group as compared to 5.2 (SE 1.123) points in the control group (Figure 6; Table 5). PMS or PMDD symptom burden also improved in both groups in the PP analysis (Multimedia Appendix 15).

**Table 5:** Estimated mean differences in PSST^a^ score between screening and month 3 for the ITT^b^ trial-2 (PMS or PMDD) analysis.

Condition	Estimated mean difference (SE)	*T* ratio (*df*=115)	*P* value
Control	–5.239 (1.123)	–4.663	<.001
Intervention	–7.080 (1.300)	–5.444	<.001

^a^PSST: Premenstrual Screening Tool.

^b^ITT: intention-to-treat.

### Secondary Outcomes

In trial 1, we observed improvements in many of our secondary outcomes after 12 weeks of using Flo in our ITT analyses. Communication and emotional score improved only in the intervention group by 0.93 points (*P*=.02). Similarly, the ratings of menstrual cycle stigma and fear of unplanned pregnancy improved by 0.61 points (*P*=.01) and 0.22 points (*P*=.04), respectively, in the intervention group only. Finally, the score of feeling of control and management of health improved in both the intervention and control groups after using Flo for 12 weeks by 1.00 (*P*<.001) and 0.45 points (*P*=.01), respectively. However, we saw no improvement in body image scores in either the intervention or control groups.

In the trial-2 ITT analysis, we observed improvements in absenteeism due to PMS or PMDD symptoms, with participants in the intervention group taking fewer absent days from work or education (estimated mean difference=–1.67, SE 0.319; *P*=.04), while no improvement was seen in the control group. We also saw no improvement in quality of life or work productivity scores in either of the trial-2 intervention or control groups.

Full ITT analysis results for trial-1 and trial-2 secondary outcomes are presented in Multimedia Appendix 16, while full PP results are presented in Multimedia Appendix 17.

## Discussion

### Principal Findings

In this study, we present the findings of 2 pilot, 2-armed RCTs on the effect of using the Flo app in (1) individuals who track their cycles and (2) individuals who track their cycles and are affected by PMS or PMDD. Our ITT analysis demonstrated improvements postintervention in all primary outcomes, namely health literacy and awareness, health and well-being, and PMS or PMDD symptom burden. As for secondary outcomes, positive effects were observed for control and management over health, communication about health, menstrual cycle stigma, and fear of unplanned pregnancies for those who track their cycles as well as absenteeism for PMS or PMDD. Quality of life, body image, and workplace productivity did not show improvement.

### Comparison With Prior Work

The primary hypothesis of this study was that using the Flo app would increase health literacy and awareness around menstrual and reproductive health. We found that menstrual (trial 1) and PMS or PMDD (trial 2) health literacy scores as well as menstrual health awareness (trial 1) significantly improved in both intervention and control groups, albeit with larger improvements in the intervention groups.

These results are consistent with preliminary observational data, which find that the Flo app can help women and people who menstruate feel more prepared throughout their menstrual cycles, helping them understand their bodies and symptoms [36,64]. These results are also consistent with previous research finding that psychoeducational and information-based interventions can improve health literacy around PMS and PMDD [37,38] and symptom scores in PMS [65]. Low health literacy has a negative impact on both individuals and health care systems alike. Poor health literacy has been associated with poorer well-being [66] and self-care [67] while contributing to higher health care costs [68], resulting from underuse of preventive services [69]. Further, the lack of knowledge and low symptom awareness often lead to delays in seeking care [70], leading to worse health outcomes. Thus, easily accessible tools addressing reproductive and menstrual health literacy have the potential to fill in the gaps in current provisioning.

Health and well-being scores (measured via a shorter version of the World Health Organization Quality of Life Brief Survey) improved in the trial-1 cycle tracking intervention group but not in the control group. PMS or PMDD symptom severity ratings (indicated by a drop in PSST scores at the final time point compared to baseline) improved in both the control and intervention groups, with a larger improvement in the intervention group. These findings are in line with previous research demonstrating that educational programs can help reduce the negative impact of menstrual and PMS or PMDD symptoms on women’s lives [71,72]. Tracking symptoms over time and identifying patterns may help individuals feel better prepared and equipped to deal with their symptoms and manage their conditions. It is also possible that the observed improvements in health and well-being may have been driven by interaction with community-based tools such as Flo’s Secret Chats, which could help individuals normalize their menstrual cycle experiences and decrease feelings of loneliness or social exclusion [29,30,33,73,74]. Improvements in PMS or PMDD symptom burden observed in the control condition could be due to participants using the app without our knowledge, motivation through exposure to study materials to think about how they may better manage any symptoms they experience, or use of other out-of-app menstrual health and PMS or PMDD resources to improve symptom management.

While the lack of existing literature on the efficacy of digital health interventions for menstrual health makes comparative analysis difficult, the magnitude of changes observed in our primary outcomes is comparable both to a previous RCT for a PMS educational app [65] and to app-based interventions for other health conditions such as sleep difficulties [75] or depression and anxiety [76,77].

In terms of secondary outcomes, questions addressing feelings of control over one’s own reproductive health showed improvements in both cycle tracking intervention and control groups, with a stronger effect observed in the intervention groups. Again, improvements in the control group are likely due to unidentified or anonymous use of the app (using the anonymous mode) by control group participants or motivation to seek out external resources and think about health management. Positive effects were observed in the cycle tracking intervention group only for outcomes including communication about health, stigma around menstrual health, and fears of unplanned pregnancies. Being able to effectively communicate with health care providers and share feelings and concerns about one’s own health are particularly important for menstrual and reproductive health. The findings from a large governmental investigation (UK Women’s Health Strategy Survey) highlighted how women and people who menstruate do not feel heard when talking to their health care provider, with 1 in 4 declaring not feeling comfortable sharing information about their menstrual cycle [12]. This could contribute to delays in diagnosis as women may not be bringing up concerning symptoms in a timely manner. Further, the finding that feelings of stigma were reduced highlights the potential of solutions like Flo app to help normalize discussions around symptoms and overall reproductive and menstrual health. Finally, fears of unplanned pregnancies may have been reduced due to the exposure to content aimed at educating users about effective contraception methods. Absenteeism was measured in the PMS or PMDD group and showed a reduced number of days taken in the intervention group only. This finding is in line with previous research highlighting the impact of conditions of the menstrual cycle on workplace productivity [36,78-81] and further suggests that psychoeducational tools may help individuals better manage their symptoms [64].

We did not observe improvements in other secondary outcomes. This could be due to a number of factors. First, while we hypothesized that better knowledge and improved management of the menstrual cycle would translate into improvements in quality of life, workplace productivity, and body image, these are complex and multifaceted outcomes. Each of these outcomes is heavily influenced by health or social and economic factors beyond those relevant to menstrual health that Flo app is not targeted to address fully. Second, while 3-month interventions tend to be the norm in the digital health field [82], quality of life, workplace productivity, and body appreciation may require longer interventions for smaller effects to be detectable and sustained over time. Finally, it has been shown that studies targeting multifactorial health behaviors tend to be less efficacious [29]. Due to this, further research, with a better-powered sample and longer timeframes, is needed to explore whether solutions like Flo app can help tackle these issues.

### Limitations and Future Research

This study does have limitations. First, as stated above, we cannot be sure that participants in the control group did not use Flo app. While our ITT analyses excluded individuals in the control condition who we identified as using the app before or during the study period, this identification was carried out using email addresses provided by study participants. If control participants signed up for the app using a different email address than the one provided to study staff, or used the Anonymous Mode feature, we would not be able to identify these individuals and exclude them on the basis of app use. Hence, any improvements in the control condition of both trials could be due to the fact that some control participants did use the Flo app, despite instructions not to. Further, participants in the control group may also have been motivated to look for other sources of knowledge on reproductive health by virtue of taking part in this study, hence improving their final scores.

Our primary findings are based on an ITT analysis, which should be considered conservative, as we included all intervention participants with some exposure to the intervention. This means that any null findings could be due to noncompliance, or low usage of the app in intervention participants. Encouragingly, however, our PP analyses do broadly show similar improvements in outcomes as in the ITT analyses.

Additionally, the intervention period was only 3 months. While this was appropriate for this kind of pilot RCT, a longer interventional period may have allowed for the detection of additional effects, particularly in the secondary and exploratory outcomes.

Furthermore, due to participant dropout, we did not achieve an adequately powered sample for the PMS or PMDD ITT analysis and all PP analyses, and therefore these results should be considered as needing further validation. Follow-up studies should investigate methods of reducing participant dropout and improving rates of completion of measures. As a remote study, some attrition is expected, but closer monitoring of participants, additional reminders or prompts to complete data collection, along with personalized and improved incentives could allow for a greater proportion of participants to fully complete the study. In-person recruitment and data collection should also be considered to aid data quality and completeness and prevent app use by participants in the control group.

Finally, the study population was limited to individuals from the United States who were English speakers and may not be fully representative of other populations worldwide. Future research should prioritize inclusion of a diverse study population by including participants from different cultural, linguistic and socioeconomic backgrounds in order to allow for robust and generalizable results.

### Conclusions

Low menstrual and reproductive health literacy and awareness can have a significant negative impact on the overall health and well-being of individuals. Further, health literacy and awareness of one’s own symptoms can greatly influence the course of conditions of the menstrual cycle and help improve knowledge of how individuals can manage them. Digital tools such as the Flo app are widely accessible and provide users with a wealth of content and tools that can be successfully turned into actionable insights. The findings from this study show how increased health literacy, symptom tracking, and exposure to a community of like-minded individuals can improve menstrual and reproductive health knowledge, symptom management, and overall health outcomes.

## Appendix

Multimedia Appendix 1 CONSORT checklist.

Multimedia Appendix 2 Trial-1 and trial-2 minimization and randomization categories.

Multimedia Appendix 3 Trial-1 and trial-2 demographic questions.

Multimedia Appendix 4 Trial 1 specific health literacy quiz questions.

Multimedia Appendix 5 Trial 2 specific health literacy quiz questions.

Multimedia Appendix 6 Trial-1 menstrual health awareness questions.

Multimedia Appendix 7 Trial-1 general health and well-being questions.

Multimedia Appendix 8 Trial-2 PMS or PMDD symptom burden (PSST score). PMDD: premenstrual dysphoric disorder; PMS: premenstrual syndrome; PSST: Premenstrual Screening Tool.

Multimedia Appendix 9 Trial-1 secondary outcomes.

Multimedia Appendix 10 Trial-2 secondary outcomes.

Multimedia Appendix 11 Flow chart of participants in the per-protocol analysis.

Multimedia Appendix 12 Per-protocol analysis demographics and baseline summary statistics for primary outcome measures.

Multimedia Appendix 13 App engagement statistics for per-protocol analyses.

Multimedia Appendix 14 Full MMRM model results for trial-1 and trial-2 intention-to-treat analyses. MMRM: Mixed Models for Repeated Measures approach.

Multimedia Appendix 15 Per-protocol estimated mean differences in primary outcome measures for trials 1 and 2.

Multimedia Appendix 16 Intention-to-treat estimated mean differences in secondary outcomes for trials 1 and 2.

Multimedia Appendix 17 Per-protocol estimated mean differences in secondary outcomes for trials 1 and 2.

## Abbreviations

CONSORT: Consolidated Standards of Reporting Trials
ITT: intention-to-treat
MMRM: Mixed Models for Repeated Measures approach
PMDD: premenstrual dysphoric disorder
PMS: premenstrual syndrome
PP: per-protocol
PSST: Premenstrual Screening Tool
RCT: randomized controlled trial
TTC: trying to conceive
