# Supplementary material for: Efficacy of the Flo App in Improving Health Literacy, Menstrual and General Health, and Well-Being in Women: Pilot Randomized Controlled Trial
Source: JMIR Mhealth Uhealth. 2024 May 2;12:e54124. doi: 10.2196/54124 (PMC11099814; doi:10.2196/54124)
Supplement: Multimedia Appendix 2 [file mhealth_v12i1e54124_app2.docx]

##### Multimedia Appendix 2. Trial 1 and Trial 2 minimisation and randomisation categories

| **Trial specific health literacy score** | '0-4', '5-8', '9-12', '13-16' |
| --- | --- |
| **Trial specific health and wellbeing score** | '0-3', '4-6', '7-9', '10-12' |
| **Age** | '18-24', '25-34', '35-44', '45-54' |
| **Yearly household income in USD** | '0-5000', '5001-10000', '10001-20000', '20001-30000', '30001-50000', '50001-75000', '75000-150000' |
| **Highest level of education completed** | categories: 'Some high school, no diploma', 'High school graduate, diploma, or the equivalent, (e.g., GED)', 'Associates degree', 'Bachelors degree', 'Masters degree', 'Doctorate degree' |
| **Race/ethnicity** | 'White, European American or Caucasian', 'Black or African American', 'Hispanic, Latina or Spanish Origin', 'Asian or Asian American', 'American Indian or Alaska Native', 'Native Hawaiian or other Pacific Islander', 'Two or more races', 'Other’ |
| **Current day of the cycle** | '0-7', '8-14', '15-21', '22-28', '+28' |
| **Reproductive health disorders** | present or absent |
| **Trial 1 (Cycle tracking) only:**  **number of children** | n |
| **Trial 1 (Cycle tracking) only:**  **number of pregnancies that did not result in a live birth** | n |
| **Trial 2 (PMS / PMDD) only:**  **PSST score** | 'Moderate', 'Severe' |

##### 
