# Supplementary material for: Efficacy of the Flo App in Improving Health Literacy, Menstrual and General Health, and Well-Being in Women: Pilot Randomized Controlled Trial
Source: JMIR Mhealth Uhealth. 2024 May 2;12:e54124. doi: 10.2196/54124 (PMC11099814; doi:10.2196/54124)
Supplement: Multimedia Appendix 3 [file mhealth_v12i1e54124_app3.docx]

##### Multimedia Appendix 3. Trial 1 and 2 Demographic Questions

1. Have you ever used the Flo app before?
   1. Yes
   2. No
2. Are you currently a US resident?
   1. Yes
   2. No
3. What is your age?
4. What is your date of birth (MM/DD/YYYY)
5. How would you describe yourself?
   1. American Indian or Alaskan Native
   2. Asian or Asian American
   3. Black or African American
   4. Hispanic, Latino, or Spanish origin
   5. Native Hawaiian or Other Pacific Islander
   6. White, European American, or Caucasian
   7. Biracial or Multiracial
   8. I prefer not to say
   9. Other (please specify)
6. What is your household income per year?
   1. Under $15,000
   2. Between $15,000 and $29,999
   3. Between $30,000 and $49,999
   4. Between $50,000 and $74,999
   5. Between $75,000 and $99,999
   6. Between $100,000 and $150,000
   7. Over $150,000
   8. I prefer not to say
7. How many people currently reside in your household?
   1. 1
   2. 2
   3. 3
   4. 4
   5. 5
   6. 6
   7. 7
   8. 8
   9. 9+
8. What is the highest level of education you’ve completed?
   1. Some high school, no diploma
   2. High school graduate, diploma, or the equivalent (e.g., GED)
   3. Associate’s degree
   4. Bachelor’s degree
   5. Master’s degree
   6. Doctorate degree
   7. I prefer not to say
   8. Other (please specify)
9. What is your gender identity? (Choose all that apply)
   1. Woman
   2. Non-binary
   3. Genderqueer or gender fluid
   4. Man
   5. Questioning or unsure
   6. Trans man
   7. Trans woman
   8. Agender
   9. I prefer not to say
   10. Additional gender category/identify not listed (Please specify)
10. Have you been diagnosed with any of the following:
    1. Polycystic ovary syndrome (PCOS)
    2. Endometriosis
    3. Uterine fibroids
    4. Ovarian cysts
    5. Ovarian cancer
    6. Adenomyosis
    7. Uterine polyps
    8. Hyperprolactinemia
    9. Pelvic inflammatory disease
    10. Thyroid disorders (hypothyroidism, hyperthyroidism, thyroid nodules)
    11. Sexually transmitted infection (STI)
    12. Infertility
    13. I have not been diagnosed with any of these conditions
    14. Other (Please specify)

*Trial 1 Specific Additional Demographic Questions*

1. How would you rate your English language proficiency?
   1. I don’t know English at all
   2. I can understand and use familiar everyday phrases and answer basic questions about personal details
   3. I can have social exchanges and give information on familiar or routine matters
   4. I can briefly describe past events and future plans, give reasons for opinions, and use the language when traveling
   5. I can communicate confidently in a variety of academic and professional environments
   6. I can use the language flexibly and accurately for social, academic, and professional purposes
   7. I can interact with ease and skill similar to a native speaker, differentiating finer shades of meaning
   8. I am an English native speaker
2. Do you have any children?
   1. Yes
   2. No
3. How many children do you have?
4. How many pregnancies have you had?
5. How many pregnancies have you had that did not result in a live birth?
6. Which of the following best describes your current reproductive health goals?
   1. I want to track my menstrual cycle and symptoms
   2. I am trying to conceive
   3. I am pregnant
   4. None of the above
7. How long have you been trying to conceive? *(Only asked to respondents who selected “I am trying to conceive” in question 6)*
   1. Less than 1 month
   2. 1-3 months
   3. 4-6 months
   4. 7 months to 1 year
   5. More than 1 year
   6. I don’t remember
8. Are you currently using any hormonal contraception (pill, injection, implant, hormonal coil)?
   1. Yes
   2. No
9. When was your last period?
   1. In the last 90 days
   2. More than 90 days ago
   3. I don’t remember
10. What week of pregnancy are you currently in? *(Only asked to respondents who selected “I am pregnant” in question 6)*

*Trial 2 Specific Additional Demographic Questions*

1. What is your employment status?
   1. Full-time
   2. Part-time
   3. Student
   4. Self-employed
   5. Unemployed
   6. Retired
2. Are you currently pregnant?
   1. Yes
   2. No
3. Are you currently taking any antidepressants?
   1. Yes
   2. No
4. Have you ever been diagnosed with a psychiatric disorder or received any treatment for a psychiatric disorder in the past? (e.g. Schizophrenia)
   1. Yes
   2. No
5. When was your last period?
   1. 0-7 days ago
   2. 8-21 days ago
   3. 21-45 days ago
   4. 45-90 days ago
   5. More than 90 days ago
6. Are you currently on any contraception that stops your period or are you willingly skipping your period via contraception? (e.g. mini pill/combined pill)
   1. Yes
   2. No
7. Are you experiencing any perimenopause or menopause symptoms? (e.g. hot flashes, night sweats, difficulty sleeping etc.)
   1. Yes
   2. No
8. Are you currently receiving any psychological or psychiatric treatment? (e.g. psychotherapy, cognitive behavioral therapy, mindfulness, meditation)
   1. Yes
   2. No
9. What is the current day of your cycle (number of days after the first day of menstruation)?
