# Supplementary material for: Efficacy of the Flo App in Improving Health Literacy, Menstrual and General Health, and Well-Being in Women: Pilot Randomized Controlled Trial
Source: JMIR Mhealth Uhealth. 2024 May 2;12:e54124. doi: 10.2196/54124 (PMC11099814; doi:10.2196/54124)
Supplement: Multimedia Appendix 4 [file mhealth_v12i1e54124_app4.docx]

##### Multimedia Appendix 4. Trial 1 Specific Health Literacy Quiz Questions

(Correct answers are in **bold**.)

1. How long does menstrual bleeding (from the first day of bleeding to the last day of bleeding) typically last?
   1. 1-2 days
   2. **3-7 days**
   3. 8-10 days
   4. I don’t know
2. How do doctors count the length of the menstrual cycle?
   1. **The length of time between the first day of the menstrual bleeding in one cycle to the first day of menstrual bleeding in the next cycle**
   2. The length of time between the first day of menstrual bleeding to the last day of menstrual bleeding
   3. The length of time between the last day of menstrual bleeding in one cycle to the last day of menstrual bleeding in the next cycle
   4. I don’t know
3. How long does a normal menstrual cycle typically last?
   1. 2-7 days
   2. 20 days or less
   3. **21-35 days**
   4. 36 days or more
   5. I don’t know
4. Which of the following should NOT be expected in a normal menstrual cycle?
   1. A period lasting 10 days
   2. Menstrual flow that affects your day-to-day life
   3. Cycle length continuously varying by 8 or more days
   4. Spotting between periods
   5. **None of the above should be expected in a normal menstrual cycle**
   6. I don’t know
5. Which of the following activities may help reduce menstrual pain?
   1. Applying heat to the area with a bath or heating pad
   2. Taking an anti-inflammatory painkiller like ibuprofen
   3. Exercising
   4. **All of the above**
   5. I don’t know
6. All of the following lifestyle changes will ease PMS symptoms EXCEPT:
   1. Regular exercise
   2. A diet that includes complex carbohydrates
   3. Decreasing consumption of caffeine and alcohol
   4. Good-quality sleep
   5. **All of the above will ease PMS symptoms**
   6. I don’t know
7. Which of these symptoms are believed to be associated with hormonal fluctuations throughout the menstrual cycle?
   1. Tender breasts
   2. Cramps
   3. Bloating
   4. Acne
   5. Cravings
   6. **All of the above are believed to be associated with hormonal changes across the cycle**
   7. I don’t know
8. Which of the following types of vaginal discharge are normal [click all that apply]?
   1. **Milky or white with no odor**
   2. Yellow
   3. White and clumpy
   4. Green
   5. All of the above are normal types of vaginal discharge
   6. I don’t know
9. Match the symptoms to the gynecological condition. [Correct matching is listed below]

| *Gynecological conditions* | *Symptoms* |
| --- | --- |
| Ovarian cysts | Bloating, pelvic pain, heaviness in the abdomen |
| Polycystic ovary syndrome (PCOS) | Irregular periods, excess testosterone, weight gain |
| Endometriosis | Severe cramps, pain during sex, excess bleeding |
| Premenstrual dysphoric disorder (PMDD) | Irritability, fatigue, extreme moodiness |

1. Which of the following is NOT an early sign of pregnancy?
   1. Missed or late period
   2. **Excessive energy**
   3. Sensitive or sore breasts
   4. Increased urination
   5. All of the above are early signs of pregnancy
   6. I don’t know
2. Approximately what percentage of women say they can have an orgasm from vaginal penetration alone?
   1. **18%**
   2. 32%
   3. 50%
   4. 77%
   5. I don’t know
3. Which of the following statements about birth control are true [click all that apply]?
   1. **Hormonal contraceptives do not protect against STIs**
   2. The copper IUD works by stopping ovulation
   3. The most effective method of birth control is the male condom
   4. All of the above are true
   5. I don’t know
