# Supplementary material for: Efficacy of the Flo App in Improving Health Literacy, Menstrual and General Health, and Well-Being in Women: Pilot Randomized Controlled Trial
Source: JMIR Mhealth Uhealth. 2024 May 2;12:e54124. doi: 10.2196/54124 (PMC11099814; doi:10.2196/54124)
Supplement: Multimedia Appendix 5 [file mhealth_v12i1e54124_app5.docx]

##### Multimedia Appendix 5. Trial 2 Specific Health Literacy Quiz Questions

(Correct answers are in **bold**.)

1. Which of the following is a PMS symptom?
   1. **Mood swings**
   2. Increased energy
   3. Redness of breasts
   4. Spotting
2. How do you know whether swollen breasts and pain are due to PMS? (check all that apply)
   1. **The symptom come before menstruation and disappear when the period begins**
   2. Pain is located at one point
   3. There are changes in appearance such as redness and bumps
   4. **The symptoms occur around your period on a regular basis**
3. To diagnose PMS, it has to meet the following criteria: (check all that apply)
   1. Bloating 5 days before the period starts
   2. **It typically ends within 4 days of the period starting**
   3. **Symptoms occur at least 3 cycles in a row**
   4. It is tied to serotonin levels
4. How many people experience PMS symptoms?
   1. Around 10%
   2. Around 20%
   3. Around 50%
   4. **Around 70%**
5. How many people experience PMDD symptoms?
   1. **At least 8%**
   2. At least 20%
   3. At least 40%
   4. At least 50%
6. What foods or drinks can make PMS symptoms worse?
   1. **Black tea**
   2. Dried fruits
   3. Avocado
   4. Bananas
7. Which of the following is true for PMS cramps? (check all that apply)
   1. **They are caused by uterine contractions before menstruation**
   2. **Applying heat to the belly can reduce pain**
   3. **Acupuncture can help relieve the pain**
   4. None of the above
8. How can you reduce PMS symptoms? (check all that apply)
   1. Use beauty products for dry skin or hair
   2. **Physical exercise**
   3. **Physical mindfulness**
   4. Have fizzy drinks
9. What can you supplement or increase intake of during PMS to reduce symptoms?
   1. **Potassium**
   2. Serotonin
   3. Vitamin D
   4. Iron
10. What usually helps with PMDD symptoms? (check all that apply)
    1. Swapping coffee for herbal tea
    2. **Talk-therapy**
    3. **Antidepressants**
    4. Fiber-rich diet
