# Supplementary material for: Efficacy of the Flo App in Improving Health Literacy, Menstrual and General Health, and Well-Being in Women: Pilot Randomized Controlled Trial
Source: JMIR Mhealth Uhealth. 2024 May 2;12:e54124. doi: 10.2196/54124 (PMC11099814; doi:10.2196/54124)
Supplement: Multimedia Appendix 6 [file mhealth_v12i1e54124_app6.docx]

##### Multimedia Appendix 6. Trial 1 Menstrual Health Awareness Questions

1. I always know when I am about to get my period. ***[scale 1 - Disagree very strongly, 7 - Agree very strongly]***
2. I understand the role of my menstrual cycle in different symptoms that I have. ***[scale 1 - Disagree very strongly, 7 - Agree very strongly]***
3. I understand how my mood and emotional state change across the menstrual cycle. ***[scale 1 - Disagree very strongly, 7 - Agree very strongly]***
4. I understand how to manage my different symptoms across my menstrual cycle. ***[scale 1 - Disagree very strongly, 7 - Agree very strongly]***
5. I understand when in my cycle I can become pregnant from sex. ***[scale 1 - Disagree very strongly, 7 - Agree very strongly]***
6. I am well-aware of different menstrual products I can use during my period. ***[scale 1 - Disagree very strongly, 7 - Agree very strongly]***
7. I am well aware of different contraception options I can use. ***[scale 1 - Disagree very strongly, 7 - Agree very strongly]***
