# Supplementary material for: Efficacy of the Flo App in Improving Health Literacy, Menstrual and General Health, and Well-Being in Women: Pilot Randomized Controlled Trial
Source: JMIR Mhealth Uhealth. 2024 May 2;12:e54124. doi: 10.2196/54124 (PMC11099814; doi:10.2196/54124)
Supplement: Multimedia Appendix 7 [file mhealth_v12i1e54124_app7.docx]

##### Multimedia Appendix 7. Trial 1 General Health and Wellbeing Questions

1. How satisfied are you with your health? ***[scale 1 - Dissatisfied very strongly, 7 - Satisfied very strongly]***
2. Over the past three months, have you experienced any physical symptoms (e.g., feeling tired or dizzy, pain, etc.) that bothered you? ***[scale 1 - Never, 7 - All of the time]***
3. Over the past three months, have you experienced any mood or mental health symptoms (e.g., feeling sad, anxious, having low self-esteem, etc.) that bothered you? ***[scale 1 - Never, 7 - All of the time]***
4. Over the past three months, have you experienced any menstrual symptoms (e.g., sore breasts, mood swings, fatigue, etc.) that bothered you? ***[scale 1 - Never, 7 - All of the time]***
5. Do you notice differences in the way your body feels in the different stages of your cycle? ***[scale 1 - Almost never true to me, 7 - Almost always true to me]***
6. How would you rate your quality of life? ***[scale 1 - Very poor, 5 - Very good]***
7. How much do you enjoy life? ***[scale 1 - Not at all, 5 - An extreme amount]***
8. To what extent do you feel your life is meaningful? ***[scale 1 - Not at all, 5 - An extreme amount]***
9. How well are you able to concentrate? ***[scale 1 - Not at all, 5 - An extreme amount]***
10. Are you able to accept your bodily appearance? ***[scale 1 - Not at all, 5 - Completely]***
11. How often do you have negative feelings such as blue mood, despair, anxiety, depression? ***[scale 1 - Never, 5 - Always]***
12. How satisfied are you with personal relationships? ***[scale 1 - Very dissatisfied, 5 - Very satisfied]***
13. How satisfied are you with your sex life? ***[scale 1 - Very dissatisfied, 5 - Very satisfied]***
14. How satisfied are you with the support you get from your friends? ***[scale 1 - Very dissatisfied, 5 - Very satisfied]***
