# Supplementary material for: Efficacy of the Flo App in Improving Health Literacy, Menstrual and General Health, and Well-Being in Women: Pilot Randomized Controlled Trial
Source: JMIR Mhealth Uhealth. 2024 May 2;12:e54124. doi: 10.2196/54124 (PMC11099814; doi:10.2196/54124)
Supplement: Multimedia Appendix 8 [file mhealth_v12i1e54124_app8.docx]

##### Multimedia Appendix 8. Trial 2 PMS/PMDD symptom burden (PSST score)

**Questions**

Do you experience some or any of the following premenstrual symptoms which start before your period and stop within a few days of bleeding? ***[scale 1 - Not at all, 5 - Severe]***

1. Anger / irritability
2. Anxiety / tension
3. Tearfulness / increased sensitivity to rejection
4. Depressed mood / hopefulness
5. Decreased interest in work activities
6. Decreased interest in home activities
7. Decreased interest in social activities
8. Difficulty concentrating
9. Fatigue / lack of energy
10. Overeating / food cravings
11. Insomnia
12. Hypersomnia (needing more sleep)
13. Feeling overwhelmed or out of control
14. Physical symptoms: breast tenderness, headaches, joint/muscle pain, bloating, weight gain

Have your symptoms, as listed above, interfered with: ***[scale 1 - Not at all, 5 - Severe]***

1. Your work efficiency or productivity
2. Your relationships with co-workers
3. Your relationships with your family
4. Your social life activities
5. Your home responsibilities
