# Supplementary material for: Efficacy of the Flo App in Improving Health Literacy, Menstrual and General Health, and Well-Being in Women: Pilot Randomized Controlled Trial
Source: JMIR Mhealth Uhealth. 2024 May 2;12:e54124. doi: 10.2196/54124 (PMC11099814; doi:10.2196/54124)
Supplement: Multimedia Appendix 9 [file mhealth_v12i1e54124_app9.docx]

##### Multimedia Appendix 9. Trial 1 Secondary Outcomes

**Control / Management over your health**

1. Over the past 6 weeks, how much have you been worried about your reproductive health? ***[scale 1 - Not at all, 7 - An extreme amount]***
2. Over the past 6 weeks, how many times have you visited a doctor for a reproductive health-related complaint?
   1. I haven’t seen a doctor in the past 6 weeks
   2. Once
   3. Twice
   4. Three times or more
3. I can tell the difference between normal menstrual cycle symptoms and those requiring medical care. ***[scale 1 - Disagree strongly, 7 - Agree strongly]***
4. I can identify when my physical, psychological, and mental health symptoms require medical care and when they don't. ***[scale 1 - Disagree strongly, 7 - Agree strongly]***

**Communication / Emotional**

1. I feel confident in my ability to communicate with my doctor about my reproductive health. ***[scale 1 - Disagree very strongly, 7 - Agree very strongly]***
2. I feel comfortable talking to my partner about my menstrual cycle. ***[scale 1 - Disagree very strongly, 7 - Agree very strongly]***
3. I feel comfortable talking to my partner about cycle-based changes in my mood. ***[scale 1 - Disagree very strongly, 7 - Agree very strongly]***
4. I feel comfortable talking to my partner about cycle-based changes in sexual desire. ***[scale 1 - Disagree very strongly, 7 - Agree very strongly]***

**Menstrual Cycle Stigma**

1. When I have my period, I am worried that someone will know. ***[scale 1 - Disagree very strongly, 7 - Agree very strongly]***
2. A menstrual cycle is something I would prefer not to have. ***[scale 1 - Disagree very strongly, 7 - Agree very strongly]***
3. Menstruating every month is a sign of a good general health. ***[scale 1 - Disagree very strongly, 7 - Agree very strongly]***

**Fear of Unplanned Pregnancy**

1. Over the past 6 weeks, have you experienced an unplanned pregnancy?
   1. Yes
   2. No
   3. I don’t know
2. How many times have you experienced an unplanned pregnancy over the past 6 weeks?
   1. 1
   2. 2
   3. 3 or more
3. Over the past 6 weeks have you feared that you were pregnant (even if you were not)? *[scale 1 - Never, 7 - Always]*

**Body Image and Appreciation**

*Standardized Body Appreciation Scale-2 (BAS-2)*

1. I respect my body ***[scale 1 - Never, 5 - Always]***
2. I feel good about my body ***[scale 1 - Never, 5 - Always]***
3. I feel that my body has at least some good qualities ***[scale 1 - Never, 5 - Always]***
4. I take a positive attitude towards my body ***[scale 1 - Never, 5 - Always]***
5. I am attentive to my body’s needs ***[scale 1 - Never, 5 - Always]***
6. I feel love for my body ***[scale 1 - Never, 5 - Always]***
7. I appreciate the different and unique characteristics of my body ***[scale 1 - Never, 5 - Always]***
8. My behavior reveals my positive attitude toward my body; for example, I walk holding my head high and smiling ***[scale 1 - Never, 5 - Always]***
9. I am comfortable in my body ***[scale 1 - Never, 5 - Always]***
10. I feel like I am beautiful even if I am different from media images of attractive people (e.g., models, actresses/actors). ***[scale 1 - Never, 5 - Always]***
