# Supplementary material for: Efficacy of the Flo App in Improving Health Literacy, Menstrual and General Health, and Well-Being in Women: Pilot Randomized Controlled Trial
Source: JMIR Mhealth Uhealth. 2024 May 2;12:e54124. doi: 10.2196/54124 (PMC11099814; doi:10.2196/54124)
Supplement: Multimedia Appendix 10 [file mhealth_v12i1e54124_app10.docx]

##### Multimedia Appendix 10. Trial 2 Secondary Outcomes

**Productivity and Absenteeism**

1. Please rate the following statements on the effect of PMS in the workplace (or school/university if you are a student). If you study and work, please refer to your main occupation when answering the questions. ***[scale 1 - Strong Disagree, 5 - Strongly Agree]***
2. Due to PMS/PMDD,, the stresses of my job were much harder to handle
3. Despite my PMS/PMDD,, I was able to finish hard tasks at work
4. My PMS/PMDD, distracted me from taking pleasure in my work
5. I felt hopeless about finishing certain tasks due to my PMS/PMDD,
6. At work, I was able to focus on achieving goals despite my PMS/PMDD,
7. Despite my PMS/PMDD,, I felt energetic enough to complete all my work

(B) How many days of absence have you taken from work or university/school in the past 3 months due to your mental health, or issues related to your menstrual cycle? If you study and work, please refer to your main occupation when answering the questions.

**Quality of life and satisfaction**

Taking everything into consideration, during the past month, how satisfied have you been with your… ***[scale 1 - Very poor, 5 - Very good]***

1. Physical health
2. Mood
3. Work
4. Household activities
5. Social relationships
6. Family relationships
7. Leisure activities
8. Ability to function in daily life
9. Sexual drive interest and/or performance
10. Economic status
11. Living / household situation
12. Ability to get around physically without feeling dizzy, unsteady or falling
13. Overall sense of wellbeing
14. Medication
15. Overall life satisfaction and contentment
