# Supplementary figures and images for: Efficacy of the Flo App in Improving Health Literacy, Menstrual and General Health, and Well-Being in Women: Pilot Randomized Controlled Trial
Source: JMIR Mhealth Uhealth. 2024 May 2;12:e54124. doi: 10.2196/54124 (PMC11099814; doi:10.2196/54124)

##### Multimedia Appendix 11: Flow chart of participants in the PP analysis

#####
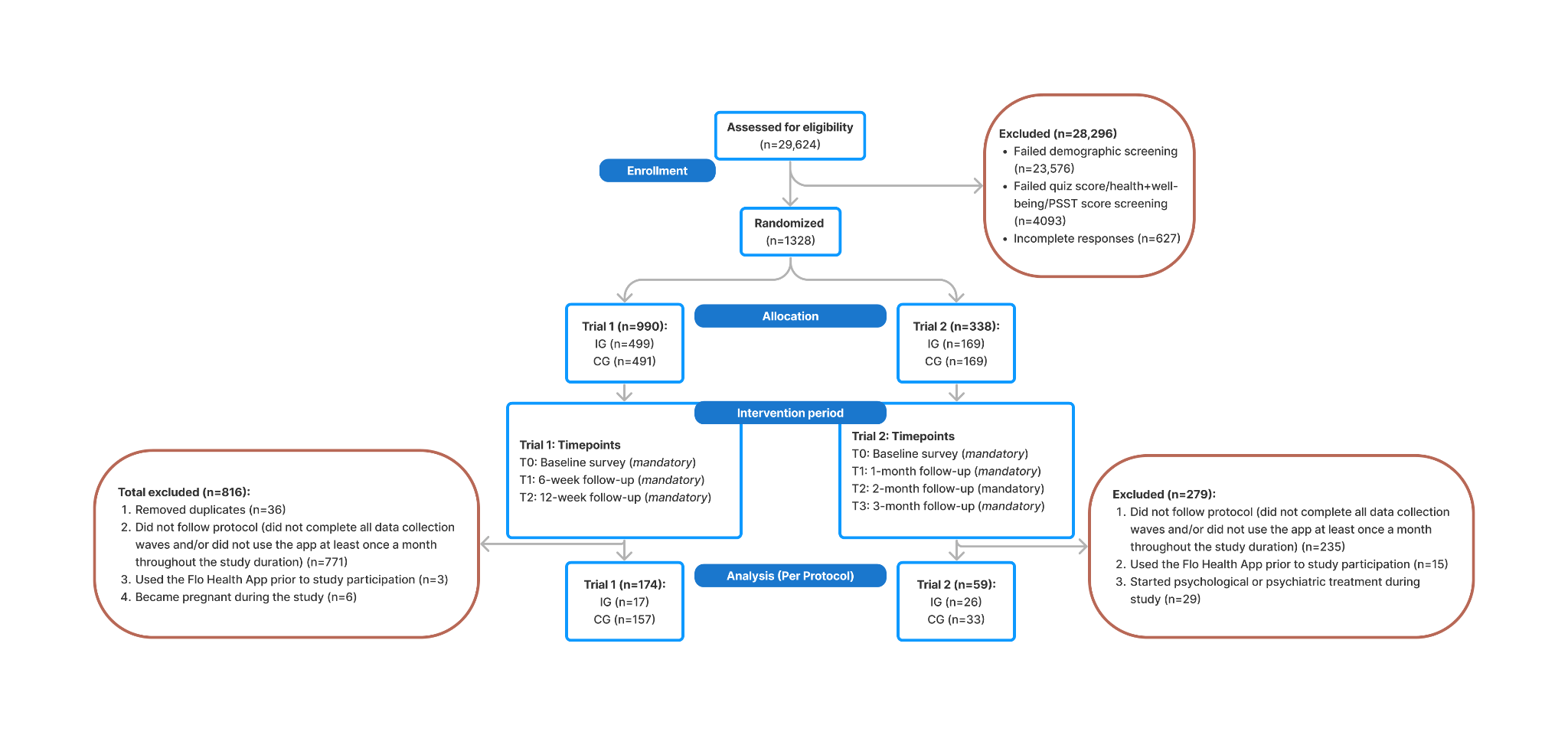


##### 

Supplement: Multimedia Appendix 11 [file mhealth_v12i1e54124_app11.docx]
