# Supplementary material for: Efficacy of the Flo App in Improving Health Literacy, Menstrual and General Health, and Well-Being in Women: Pilot Randomized Controlled Trial
Source: JMIR Mhealth Uhealth. 2024 May 2;12:e54124. doi: 10.2196/54124 (PMC11099814; doi:10.2196/54124)
Supplement: Multimedia Appendix 12 [file mhealth_v12i1e54124_app12.docx]

##### Multimedia Appendix 12. PP analysis demographics and baseline summary statistics for primary outcome measures

|  | **Cycle Tracking** | | | | **PMS/PMDD** | | | |
| --- | --- | --- | --- | --- | --- | --- | --- | --- |
|  | **Control (N=157)** | **Intervention (N=17)** | **Total (N=174)** | **p value** | **Control (N=33)** | **Intervention (N=26)** | **Total (N=59)** | **p value** |
| **Age Group** |  |  |  | 0.838^1^ |  |  |  | 0.084^1^ |
| 18-24 | 13  (8.3%) | 2  (11.8%) | 15  (8.6%) |  | 6 (18.2%) | 5 (19.2%) | 11 (18.6%) |  |
| 25-34 | 74 (47.1%) | 7  (41.2%) | 81 (46.6%) |  | 5 (15.2%) | 13 (50.0%) | 18 (30.5%) |  |
| 35-44 | 70 (44.6%) | 8  (47.1%) | 78 (44.8%) |  | 18 (54.5%) | 6 (23.1%) | 24 (40.7%) |  |
| 45-54 | 0  (0.0%) | 0  (0.0%) | 0  (0.0%) |  | 4 (12.1%) | 2 (7.7%) | 6 (10.2%) |  |
| **Race/ethnicity** |  |  |  | 0.428^1^ |  |  |  | 0.871^2^ |
| American Indian or Alaskan Native | 2  (1.3%) | 1  (5.9%) | 3  (1.7%) |  | 0  (0.0%) | 1  (3.0%) | 1  (1.3%) |  |
| Asian or Asian American | 8  (5.1%) | 2  (11.8%) | 10  (5.7%) |  | 2 (6.1%) | 2 (7.7%) | 4 (6.8%) |  |
| Biracial or Multiracial | 8  (5.1%) | 0  (0.0%) | 8  (4.6%) |  | 2 (6.1%) | 2 (7.7%) | 4 (6.8%) |  |
| Black or African American | 24 (15.3%) | 0  (0.0%) | 24 (13.8%) |  | 4 (12.1%) | 2 (7.7%) | 6 (10.2%) |  |
| Hispanic, Latino, or Spanish origin | 13  (8.3%) | 2  (11.8%) | 15  (8.6%) |  | 5 (15.2%) | 2 (7.7%) | 7 (11.9%) |  |
| Native Hawaiian or Other Pacific Islander | 1  (0.6%) | 0  (0.0%) | 1  (0.6%) |  | 0  (0.0%) | 0  (0.0%) | 0  (0.0%) |  |
| White, European American, or Caucasian | 99 (63.1%) | 12  (70.6%) | 111 (63.8%) |  | 20 (60.6%) | 18 (69.2%) | 38 (64.4%) |  |
| Other (please specify) | 2  (1.3%) | 0  (0.0%) | 2  (1.1%) |  | 0  (0.0%) | 0  (0.0%) | 0  (0.0%) |  |
| **Household Income** |  |  |  | 0.198^1^ |  |  |  | 0.903^1^ |
| Under $15,000 | 25 (15.9%) | 2  (11.8%) | 27 (15.5%) |  | 4 (12.1%) | 3 (11.5%) | 7 (11.9%) |  |
| Between $15,000 and $29,999 | 29 (18.5%) | 1  (5.9%) | 30 (17.2%) |  | 2 (6.1%) | 5 (19.2%) | 7 (11.9%) |  |
| Between $30,000 and $49,999 | 37 (23.6%) | 6  (35.3%) | 43 (24.7%) |  | 8 (24.2%) | 5 (19.2%) | 13 (22.0%) |  |
| Between $50,000 and $74,999 | 32 (20.4%) | 3  (17.6%) | 35 (20.1%) |  | 10 (30.3%) | 6 (23.1%) | 16 (27.1%) |  |
| Between $75,000 and $99,999 | 23 (14.6%) | 1  (5.9%) | 24 (13.8%) |  | 5 (15.2%) | 2 (7.7%) | 7 (11.9%) |  |
| Between $100,000 and $150,000 | 5  (3.2%) | 3  (17.6%) | 8  (4.6%) |  | 4 (12.1%) | 3 (11.5%) | 7 (11.9%) |  |
| Over $150,000 | 6  (3.8%) | 1  (5.9%) | 7  (4.0%) |  | 0 (0.0%) | 2 (7.7%) | 2 (3.4%) |  |
| **Highest Education Level** |  |  |  | 0.006^1^ |  |  |  | 0.610^1^ |
| N-Miss | 1 | 0 | 1 |  | 0 | 0 | 0 |  |
| Incomplete or complete secondary education | 77 (49.4%) | 3  (17.6%) | 80 (46.2%) |  | 12 (36.4%) | 10 (38.5%) | 22 (37.3%) |  |
| Some post secondary education, certificate, or associate’s degree | 27 (17.3%) | 3  (17.6%) | 30 (17.3%) |  | 5 (15.2%) | 6 (23.1%) | 11 (18.6%) |  |
| Bachelor’s degree or further education | 52 (33.3%) | 11  (64.7%) | 63 (36.4%) |  | 16 (48.5%) | 10 (38.5%) | 26 (44.1%) |  |
| **Health Literacy** |  |  |  | 0.470^3^ |  |  |  | 0.063^3^ |
| Mean (SD) | 9.178 (2.217) | 9.588 (2.238) | 9.218 (2.216) |  | 9.000 (2.107) | 9.962 (1.685) | 9.424 (1.976) |  |
| Range | 3.000 - 14.000 | 4.000 - 13.000 | 3.000 - 14.000 |  | 6.000 - 12.000 | 6.000 - 12.000 | 6.000 - 12.000 |  |
| **Menstrual Awareness** |  |  |  | 0.991^3^ |  |  |  | NA |
| Mean (SD) | 34.783 (6.278) | 34.765 (7.093) | 34.782 (6.340) |  | NA | NA | NA |  |
| Range | 13.000 - 49.000 | 23.000 - 48.000 | 13.000 - 49.000 |  | NA | NA | NA |  |
| **Health and Wellbeing** |  |  |  | 0.443^3^ |  |  |  | NA |
| Mean (SD) | 44.847 (10.590) | 46.882 (7.817) | 45.046 (10.351) |  | NA | NA | NA |  |
| Range | 16.000 - 67.000 | 29.000 - 59.000 | 16.000 - 67.000 |  | NA | NA | NA |  |
| **PSST Score** |  |  |  | NA |  |  |  | 0.024^3^ |
| Mean (SD) | NA | NA | NA |  | 33.030 (8.483) | 37.885 (7.290) | 35.169 (8.278) |  |
| Range | NA | NA | NA |  | 17.000 - 54.000 | 24.000 - 53.000 | 17.000 - 54.000 |  |
| 1. Trend test for ordinal variables  2. Pearson's Chi-squared test  3. Linear Model ANOVA | | | | | | | | |

##### 
