# Supplementary material for: Efficacy of the Flo App in Improving Health Literacy, Menstrual and General Health, and Well-Being in Women: Pilot Randomized Controlled Trial
Source: JMIR Mhealth Uhealth. 2024 May 2;12:e54124. doi: 10.2196/54124 (PMC11099814; doi:10.2196/54124)
Supplement: Multimedia Appendix 13 [file mhealth_v12i1e54124_app13.docx]

##### Multimedia Appendix 13: App engagement statistics for PP analyses

#####

|  | **Trial 1 (Cycle Tracking)** | **Trial 2 (PMS/PMDD)** |
| --- | --- | --- |
| **Session Length (Seconds)** |  |  |
| Mean (SD) | 234.449 (181.982) | 156.234 (145.433) |
| **Total Sessions** |  |  |
| Mean (SD) | 29.938 (20.939) | 70.885 (53.833) |
| **Active Days** |  |  |
| Mean (SD) | 15.438 (10.954) | 38.077 (26.595) |

##### 
