# Supplementary material for: Efficacy of the Flo App in Improving Health Literacy, Menstrual and General Health, and Well-Being in Women: Pilot Randomized Controlled Trial
Source: JMIR Mhealth Uhealth. 2024 May 2;12:e54124. doi: 10.2196/54124 (PMC11099814; doi:10.2196/54124)
Supplement: Multimedia Appendix 14 [file mhealth_v12i1e54124_app14.docx]

##### Multimedia Appendix 14: Full MMRM model results for Trial 1 and 2 ITT analyses

Table S1. Full MMRM results for Cycle Tracking ITT Health Literacy Scores (Trial 1)

|  | **Estimate** | **Std. Error** | **df** | **t value** | **Pr(>\|t\|)** |
| --- | --- | --- | --- | --- | --- |
| (Intercept) | 8.134 | 0.473 | 318.763 | 17.206 | 0.000 |
| Age Group | -0.100 | 0.176 | 308.000 | -0.567 | 0.571 |
| Education Group | 0.488 | 0.141 | 308.000 | 3.475 | 0.001 |
| Household Income | 0.080 | 0.077 | 308.000 | 1.034 | 0.302 |
| Group (Intervention) | -0.134 | 0.242 | 309.302 | -0.555 | 0.579 |
| Timepoint (12 weeks) | 0.523 | 0.173 | 311.000 | 3.026 | 0.003 |
| Group (Intervention) : Timepoint (12 weeks) | 0.585 | 0.259 | 311.000 | 2.255 | 0.025 |
|  | **Baseline** | **12 Weeks** |  |  |  |
| Baseline | 4.494 | 2.530 |  |  |  |
| 12 Weeks | 2.530 | 5.766 |  |  |  |

Table S2. Full MMRM model results for PMS/PMDD ITT Health Literacy Scores (Trial 2)

|  | **Estimate** | **Std. Error** | **df** | **t value** | **Pr(>\|t\|)** |
| --- | --- | --- | --- | --- | --- |
| (Intercept) | 8.015 | 0.742 | 113.015 | 10.808 | 0 |
| Age Group | 0.049 | 0.209 | 112 | 0.232 | 0.817 |
| Education Group | 0.43 | 0.21 | 112 | 2.049 | 0.043 |
| Household Income | -0.04 | 0.124 | 112 | 0.326 | 0.745 |
| Group (Intervention) | 0.819 | 0.359 | 111.655 | 2.279 | 0.025 |
| Timepoint (Month 3) | 0.642 | 0.276 | 115 | 2.327 | 0.022 |
| Group (Intervention) : Timepoint (Month 3) | 0.558 | 0.422 | 115 | 1.323 | 0.188 |
|  | **Pre Screening** | **Month 3** |  |  |  |
| Pre Screening | 3.66 | 2.684 |  |  |  |
| Month 3 | 2.684 | 6.903 |  |  |  |

Table S3. Full MMRM model results for ITT menstrual health awareness scores (Trial 1)

|  | **Estimate** | **Std. Error** | **df** | **t value** | **Pr(>\|t\|)** |
| --- | --- | --- | --- | --- | --- |
| (Intercept) | 32.569 | 1.277 | 328.314 | 25.504 | 0.000 |
| Age Group | 1.053 | 0.471 | 308 | 2.237 | 0.026 |
| Education Group | 0.302 | 0.207 | 308 | 1.457 | 0.146 |
| Household Income | -0.703 | 0.377 | 308 | -1.866 | 0.063 |
| Group (Intervention) | -0.024 | 0.693 | 310.029 | -0.035 | 0.972 |
| Timepoint (12 weeks) | 1.845 | 0.461 | 311 | 4.005 | 0.000 |
| Group (Intervention) : Timepoint (12 weeks) | 2.126 | 0.691 | 311 | 3.076 | 0.002 |
|  | **Baseline** | **12 Weeks** |  |  |  |
| Baseline | 37.006 | 17.714 |  |  |  |
| 12 Weeks | 17.714 | 35.336 |  |  |  |

Table S4. Full MMRM Model results for ITT general health and wellbeing scores (Trial 1)

|  | **Estimate** | **Std. Error** | **df** | **t value** | **Pr(>\|t\|)** |
| --- | --- | --- | --- | --- | --- |
| (Intercept) | 37.816 | 2.180 | 315.620 | 17.349 | 0.000 |
| Age Group | 0.449 | 0.813 | 308.087 | 0.553 | 0.581 |
| Education Group | 0.694 | 0.650 | 308.056 | 1.068 | 0.286 |
| Household Income | 1.444 | 0.358 | 308.047 | 4.033 | 0.000 |
| Group (Intervention) | 0.945 | 1.090 | 308.191 | 0.867 | 0.387 |
| Timepoint (6 weeks) | 0.016 | 0.488 | 298.449 | 0.034 | 0.973 |
| Timepoint (12 weeks) | 0.764 | 0.517 | 310.988 | 1.478 | 0.140 |
| Group (Intervention) : Timepoint (6 weeks) | 1.618 | 0.736 | 300.466 | 2.198 | 0.029 |
| Group (Intervention) : Timepoint (12 weeks) | 2.674 | 0.776 | 310.988 | 3.447 | 0.001 |
|  | **Pre-screen** | **6 weeks** | **12 weeks** |  |  |
| **Pre-screen** | 91.345 | 76.098 | 68.683 |  |  |
| **6 weeks** | 76.098 | 100.021 | 82.163 |  |  |
| **12 weeks** | 68.683 | 82.163 | 92.541 |  |  |

Table S5. Full MMRM Model results for ITT PMS/PMDD symptom burden (Trial 2)

|  | **Estimate** | **Std. Error** | **df** | **t value** | **Pr(>\|t\|)** |
| --- | --- | --- | --- | --- | --- |
| (Intercept) | 36.414 | 3.256 | 114.573 | 11.185 | 0.000 |
| Age Group | 0.081 | 0.914 | 112.251 | 0.089 | 0.929 |
| Education Group | -1.49 | 0.918 | 112.02 | -1.624 | 0.107 |
| Household Income | -0.118 | 0.541 | 112.082 | -0.219 | 0.827 |
| Group (Intervention) | 4.094 | 1.637 | 112.328 | 2.502 | 0.014 |
| Timepoint (Month 1) | -4.264 | 1.022 | 112.137 | -4.174 | 0.000 |
| Timepoint (Month 2) | -5.133 | 1.208 | 111.515 | -4.248 | 0.000 |
| Timepoint (Month 3) | -5.239 | 1.123 | 115.012 | -4.663 | 0.000 |
| Group (Intervention) :Timepoint (Month 1) | -0.48 | 1.548 | 110.732 | -0.31 | 0.757 |
| Group (Intervention) : Timepoint (Month 2) | -0.598 | 1.841 | 110.935 | -0.325 | 0.746 |
| Group (Intervention) : Timepoint (Month 3) | -1.841 | 1.719 | 115.012 | -1.071 | 0.286 |
|  | **Pre-screen** | **Month 1** | **Month 2** | **Month 3** |  |
| **Pre-screen** | 75.342 | 59.328 | 50.113 | 51.762 |  |
| **Month 1** | 59.328 | 109.168 | 81.264 | 77.33 |  |
| **Month 2** | 50.113 | 81.264 | 115.948 | 80.129 |  |
| **Month 3** | 51.762 | 77.33 | 80.129 | 112.745 |  |
