# Supplementary material for: Efficacy of the Flo App in Improving Health Literacy, Menstrual and General Health, and Well-Being in Women: Pilot Randomized Controlled Trial
Source: JMIR Mhealth Uhealth. 2024 May 2;12:e54124. doi: 10.2196/54124 (PMC11099814; doi:10.2196/54124)
Supplement: Multimedia Appendix 15 [file mhealth_v12i1e54124_app15.docx]

##### Multimedia Appendix 15. PP estimated mean differences in primary outcome measures for Trial 1 and Trial 2.

| **Trial** | **Outcome Measure** | **Control or Intervention** | **Est. mean difference** | **SE** | **df** | **T ratio** | **P value** |
| --- | --- | --- | --- | --- | --- | --- | --- |
| Trial 1 (Cycle Tracking) | Menstrual Health Literacy | Control | 0.429 | 0.189 | 171 | 2.273 | 0.024 |
| Trial 1 (Cycle Tracking) | Menstrual Health Literacy | Intervention | 0.588 | 0.572 | 171.0 | 1.028 | 0.306 |
| Trial 2 (PMS/PMDD) | PMS/PMDD Health Literacy | Control | 0.424 | 0.371 | 57 | 1.143 | 0.258 |
| Trial 2 (PMS/PMDD) | PMS/PMDD Health Literacy | Intervention | 1.731 | 0.418 | 57 | 4.139 | <0.001 |
| Trial 1 (Cycle Tracking) | Menstrual Health Awareness | Control | 1.628 | 0.455 | 171 | -3.578 | <0.001 |
| Trial 1 (Cycle Tracking) | Menstrual Health Awareness | Intervention | 3.412 | 1.379 | 171 | -2.475 | 0.014 |
| Trial 1 (Cycle Tracking) | Health and Wellbeing | Control | 0.929 | 0.485 | 171 | -1.917 | 0.057 |
| Trial 1 (Cycle Tracking) | Health and Wellbeing | Intervention | 3.353 | 1.469 | 171 | -2.283 | 0.024 |
| Trial 2 (PMS/PMDD) | PSST Score | Control | -5.242 | 1.651 | 57 | -3.175 | 0.002 |
| Trial 2 (PMS/PMDD) | PSST Score | Intervention | -5.885 | 1.860 | 57 | -3.164 | 0.002 |
