# Supplementary material for: Efficacy of the Flo App in Improving Health Literacy, Menstrual and General Health, and Well-Being in Women: Pilot Randomized Controlled Trial
Source: JMIR Mhealth Uhealth. 2024 May 2;12:e54124. doi: 10.2196/54124 (PMC11099814; doi:10.2196/54124)
Supplement: Multimedia Appendix 16 [file mhealth_v12i1e54124_app16.docx]

##### Multimedia Appendix 16. ITT estimated mean differences in secondary outcomes for trials 1 and 2.

| **Trial** | **Outcome Measure** | **Control or Intervention** | **Est. mean difference** | **SE** | **df** | **T ratio** | **P value** |
| --- | --- | --- | --- | --- | --- | --- | --- |
| Trial 1 (Cycle Tracking) | Communication and emotion | Control | 0.379 | 0.344 | 311.000 | 1.101 | 0.272 |
| Trial 1 (Cycle Tracking) | Communication and emotion | Intervention | 0.928 | 0.385 | 311.000 | 2.409 | 0.017 |
| Trial 1 (Cycle Tracking) | Menstrual Stigma | Control | 0.132 | 0.201 | 311.004 | 0.659 | 0.510 |
| Trial 1 (Cycle Tracking) | Menstrual Stigma | Intervention | -0.612 | 0.224 | 311.004 | -2.725 | 0.007 |
| Trial 1 (Cycle Tracking) | Unplanned Pregnancy Fear | Control | -0.115 | 0.092 | 311.001 | -1.256 | 0.210 |
| Trial 1 (Cycle Tracking) | Unplanned Pregnancy Fear | Intervention | -0.216 | 0.102 | 311.001 | -2.108 | 0.036 |
| Trial 1 (Cycle Tracking) | Control and Management of Health | Control | 0.454 | 0.177 | 311.002 | 2.563 | 0.011 |
| Trial 1 (Cycle Tracking) | Control and Management of Health | Intervention | 1.007 | 0.198 | 311.002 | 5.082 | 0.000 |
| Trial 1 (Cycle Tracking) | Body Image | Control | 0.316 | 0.466 | 311.015 | 0.679 | 0.498 |
| Trial 1 (Cycle Tracking) | Body Image | Intervention | 0.755 | 0.521 | 311.015 | 1.450 | 0.148 |
| Trial 2 (PMS/PMDD) | Absenteeism | Control | -0.72 | 0.594 | 144 | -1.211 | 0.228 |
| Trial 2 (PMS/PMDD) | Absenteeism | Intervention | -1.672 | 0.673 | 144 | -2.485 | 0.014 |
| Trial 2 (PMS/PMDD) | Productivity (SPS-6) | Control | -0.427 | 0.375 | 143.997 | -1.14 | 0.256 |
| Trial 2 (PMS/PMDD) | Productivity (SPS-6) | Intervention | 0.203 | 0.424 | 143.997 | 0.479 | 0.633 |
| Trial 2 (PMS/PMDD) | Quality of Life | Control | 0.634 | 1.042 | 144 | 0.609 | 0.544 |
| Trial 2 (PMS/PMDD) | Quality of Life | Intervention | -0.062 | 1.179 | 144 | -0.053 | 0.958 |

##### 
