# Supplementary material for: Efficacy of the Flo App in Improving Health Literacy, Menstrual and General Health, and Well-Being in Women: Pilot Randomized Controlled Trial
Source: JMIR Mhealth Uhealth. 2024 May 2;12:e54124. doi: 10.2196/54124 (PMC11099814; doi:10.2196/54124)
Supplement: Multimedia Appendix 17 [file mhealth_v12i1e54124_app17.docx]

##### Multimedia Appendix 17. PP estimated mean differences in secondary outcomes for trial 1 and trial 2

| **Trial** | **Outcome Measure** | **Control or Intervention** | **Est. mean difference** | **SE** | **df** | **T ratio** | **P value** |
| --- | --- | --- | --- | --- | --- | --- | --- |
| Trial 1 (Cycle Tracking) | Communication and emotion | Control | 0.263 | 0.377 | 171.008 | 0.698 | 0.486 |
| Trial 1 (Cycle Tracking) | Communication and emotion | Intervention | 1.118 | 1.141 | 171.008 | 0.980 | 0.329 |
| Trial 1 (Cycle Tracking) | Menstrual Stigma | Control | 0.083 | 0.192 | 171.002 | 0.434 | 0.665 |
| Trial 1 (Cycle Tracking) | Menstrual Stigma | Intervention | -2.765 | 0.581 | 171.002 | -4.754 | 0.000 |
| Trial 1 (Cycle Tracking) | Unplanned Pregnancy Fear | Control | -0.160 | 0.081 | 171.000 | 1.968 | 0.051 |
| Trial 1 (Cycle Tracking) | Unplanned Pregnancy Fear | Intervention | -0.353 | 0.247 | 171.000 | -1.431 | 0.154 |
| Trial 1 (Cycle Tracking) | Control and Management of Health | Control | 0.417 | 0.186 | 171.003 | 2.235 | 0.027 |
| Trial 1 (Cycle Tracking) | Control and Management of Health | Intervention | 1.235 | 0.565 | 171.003 | 2.188 | 0.030 |
| Trial 1 (Cycle Tracking) | Body Image | Control | -0.071 | 0.436 | 171.002 | -0.162 | 0.872 |
| Trial 1 (Cycle Tracking) | Body Image | Intervention | 1.529 | 1.321 | 171.002 | 1.158 | 0.248 |
| Trial 2 (PMS/PMDD) | Absenteeism | Control | 0.031 | 0.354 | 56 | 0.088 | 0.930 |
| Trial 2 (PMS/PMDD) | Absenteeism | Intervention | 0.654 | 0.393 | 56 | 1.664 | 0.102 |
| Trial 2 (PMS/PMDD) | SPS-6 | Control | 0.576 | 0.568 | 57.008 | 1.014 | 0.315 |
| Trial 2 (PMS/PMDD) | SPS-6 | Intervention | 0.000 | 0.639 | 57.008 | 0.000 | 1.000 |
| Trial 2 (PMS/PMDD) | QOL | Control | -0.848 | 1.761 | 56.997 | -0.482 | 0.632 |
| Trial 2 (PMS/PMDD) | QOL | Intervention | -1.692 | 1.984 | 56.997 | -0.853 | 0.397 |
